# Response and resilience of karst subterranean estuary communities to precipitation impacts

**DOI:** 10.1002/ece3.10415

**Published:** 2023-08-14

**Authors:** Fernando Calderón Gutiérrez, Thomas M. Iliffe, Elizabeth Borda, Germán Yáñez Mendoza, Jessica Labonté

**Affiliations:** ^1^ Department of Marine Biology Texas A&M University at Galveston Galveston Texas USA; ^2^ Department of Natural Sciences Texas A&M University San Antonio San Antonio Texas USA; ^3^ Circulo Espeleológico del Mayab A.C. Cozumel Mexico; ^4^ Galveston Texas USA

**Keywords:** anchialine, cave, community structure, precipitation impacts, stygobiont, temperature shift

## Abstract

The impact of meteorological phenomena on ecosystem communities of karst subterranean estuaries (KSEs) remains unknown. KSEs are characterized by vertically stratified groundwater separated by a halocline and host endemic aquatic cave‐adapted fauna (stygobionts). In October 2015, 8 days of heavy precipitation caused the first recorded mortality event in the KSE. This event was marked by a halocline shift 5 m deeper. The present study aimed to provide insights into resilience of KSEs faunal communities to temporal shifts in temperature and precipitation. Cave water temperature decreased on average 0.0068°C per mm of accumulated precipitation over 4 days, which can add up to, and surpass, the interannual temperature variation in cases of heavy precipitations. Biological surveys (2012–2021) conducted within cave systems El Aerolito and La Quebrada, in Cozumel, indicated that change in community structure was not detected and stygobionts were resilient; however, marine species inhabiting the caves were impacted. Overall, the faunal community at KSEs remains resilient within short‐term meteorological phenomena despite shifts of non‐stygobionts.

## Graphical abstract

Heavy precipitation events caused a halocline shift resulting in a mortality event at a karst subterranean estuary, affecting the fauna inhabiting under the halocline (dotted white line) at the marine groundwater layer (a‐b). Overall, marine species (c‐e) inhabiting the caves were impacted, and cave‐adapted fauna (f‐i) remain resilient to short‐term meteorological phenomena.

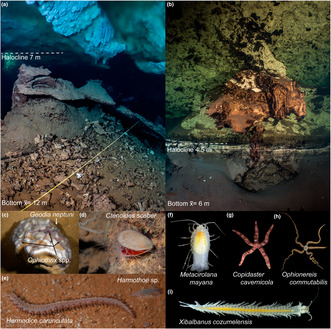

## Resumen

El impacto de fenómenos meteorológicos en las comunidades de los ecosistemas de estuarios subterráneos kársticos (KSE, por sus siglas en inglés) sigue siendo desconocido. Los KSE se caracterizan por aguas subterráneas estratificadas verticalmente separadas por una haloclina, y albergan fauna acuática endémica adaptada a la vida en cuevas (estigobiontes). En octubre de 2015, ocho días de fuertes precipitaciones causaron el primer evento de mortandad registrado en un KSE. Este evento estuvo marcado por un desplazamiento de la haloclina, resultando en una haloclina 5 m más profunda. El presente estudio tuvo como objetivo proveer información sobre la resiliencia de las comunidades faunísticas de los KSE a cambios temporales en la temperatura y la precipitación. La temperatura del agua de la cueva disminuyó en promedio 0.0068°C por mm de precipitación acumulado durante cuatro días, lo cual puede alcanzar, e incluso superar, la variación interanual de la temperatura debido a fuertes precipitaciones. Los estudios biológicos (2012–2021) realizados dentro de los sistemas de cuevas El Aerolito y La Quebrada, en Cozumel, indicaron que no se detectó un cambio en la estructura de la comunidad y que los estigobiontes fueron resilientes, sin embargo, las especies marinas que habitan las cuevas se vieron afectadas. En general, la comunidad de fauna de KSEs se mantiene resiliente a los fenómenos meteorológicos a corto plazo a pesar de los cambios en la fauna no estigobia.

## 1 BACKGROUND

**FIGURE 1:**
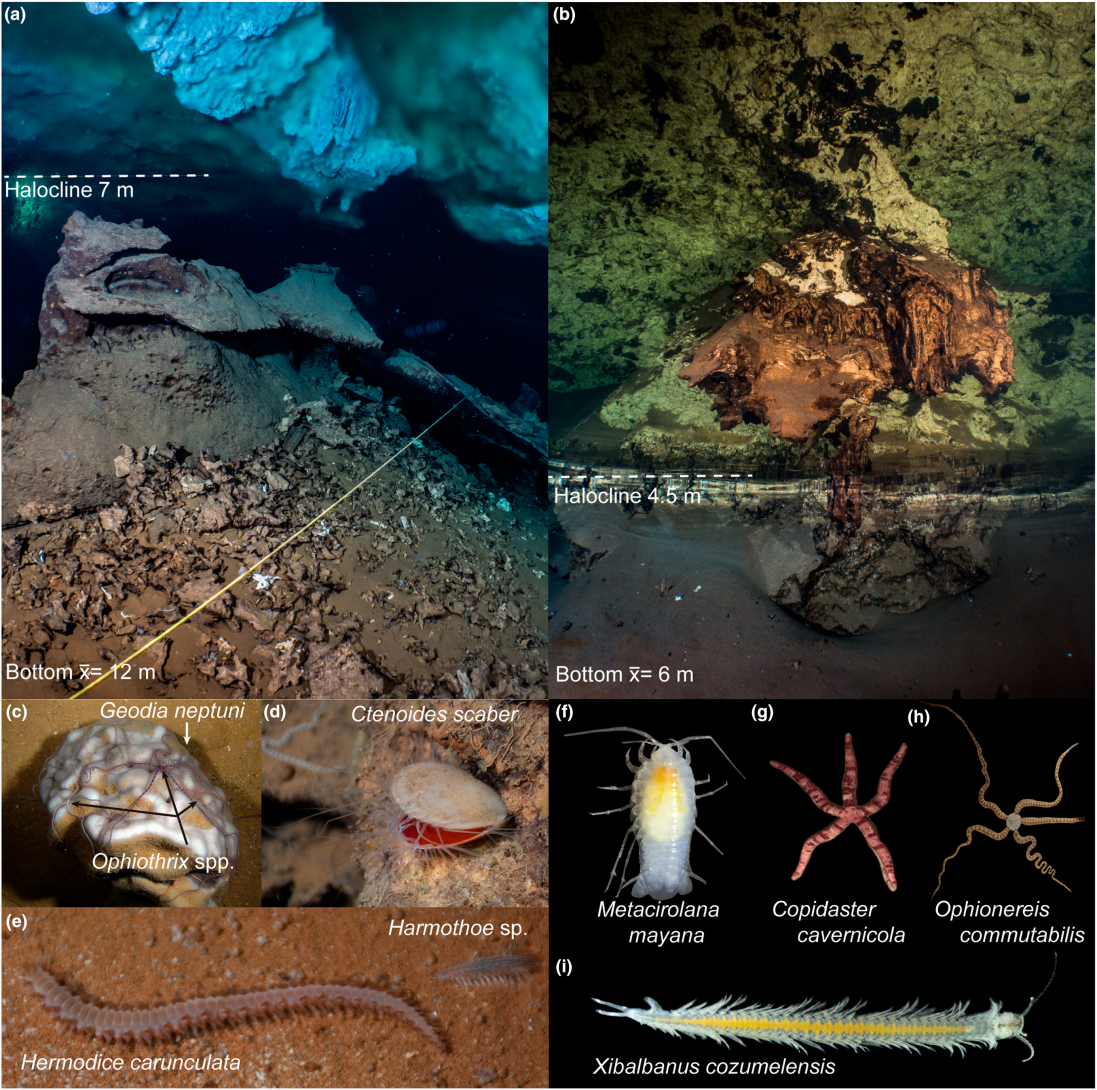
Karst subterranean estuaries and examples of the fauna inhabiting them. (a) El Aerolito and (b) La Quebrada; white dashed line indicates the position of the halocline. (c–e) examples of stygophiles (i.e., species with open ocean and cave distributions). (f–i) examples of stygobionts (i.e., endemic aquatic cave‐adapted fauna). Photo credits: (a, b) Laurent Miroult; (c–i) Fernando Calderón Gutiérrez.

**FIGURE 2:**
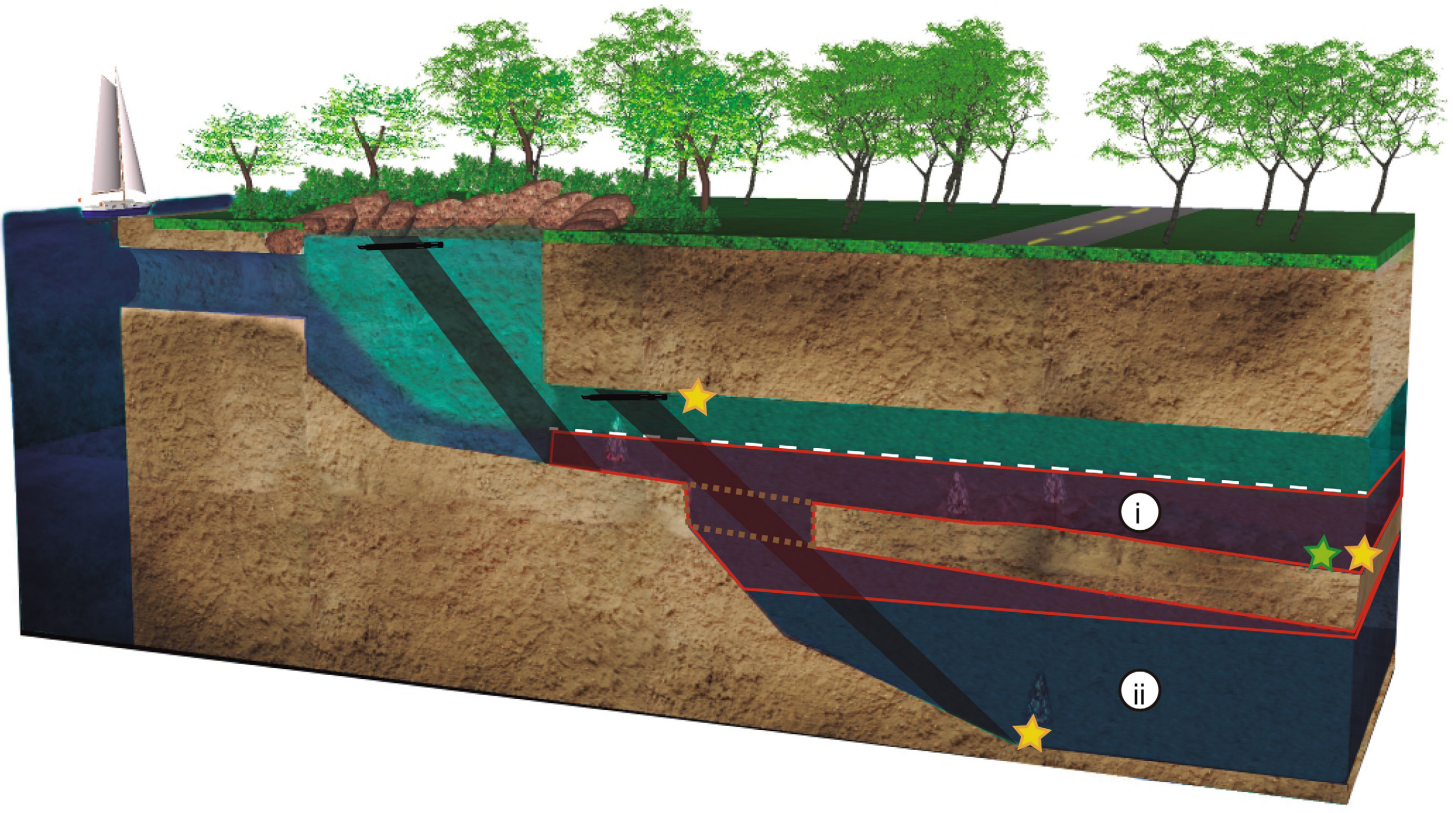
Diagram displaying a cross‐section of the KSE (not to scale). The main difference between El Aerolito and La Quebrada is that the latter does not have a deeper section (brown rectangle with dotted line represents the bottom at La Quebrada), and the halocline is closest to the bottom at La Quebrada. (i) The main cave passage showing the position of the halocline (white dashed line); macroinvertebrates were dominant below the halocline (marine groundwater). Shaded in red, mortality event zone, showing the position of the halocline 5 m deeper in October 2015. (ii) Deeper cave section (only at El Aerolito) showing displaced halocline position; migration of fauna recorded from the impacted region (i) was not observed. Stars indicate the position of temperature data loggers (El Aerolito at 5, 11, and 18 m; La Quebrada at 5 m). Black shaded areas with black multiparameter sonde on top indicate where the hydrogeological profiles were measured. Image modified from Calderón‐Gutiérrez et al. (2018).

In October 2015, a high mortality event of macroinvertebrates was recorded for the first time in a karst subterranean estuary (KSE; Calderón‐Gutiérrez et al., 2018). KSEs are coastal ecosystems characterized by vertically stratified groundwater, where one or more layers of fresh (i.e., meteoric lens) to brackish water (i.e., mixing zone) are buoyed over marine groundwater, each separated by a halocline interface (Figure 1a,b; Bishop et al., 2015; Brankovits et al., 2018). Biological surveys of the cave system El Aerolito (Cozumel Island, Mexico) (2011–2016) showed macroinvertebrates, comprised of stygoxenes (i.e., accidental cave fauna), stygophiles (i.e., species with open ocean and cave distributions; Figure 1c–e), and stygobionts (i.e., cave‐adapted endemics; Figure 1f–i) as dominant within the marine groundwater layer, below the halocline (Calderón‐Gutiérrez et al., 2018). After 8 days of heavy precipitation (October 13–20, 2015), the halocline adjacent to the marine groundwater was displaced 5 m deeper (Calderón‐Gutiérrez et al., 2018), resulting in a mortality event (Figure 2). Migration of fauna from the impacted region into deeper sections was not observed. A video of this mortality event is available as part of the supplemental material of Calderón‐Gutiérrez et al. (2018). Similar events have not been observed since Hurricane Wilma in 2005 (G.Y.M. personal observation as a frequent diver of El Aerolito since the late 1980s).

KSEs are characterized by high levels of micro‐endemic stygobiont species (limited to one or two caves) with small populations (Calderón‐Gutiérrez et al., 2018; Mammola, Cardoso, et al., 2019), with some higher taxa (e.g., class Remipedia; Bishop et al., 2015) only represented by stygobionts. KSEs with direct connection with the ocean show tidal and interannual variation in temperature and salinity. As such, these caves appear to be more susceptible to external disturbances as opposed to caves with indirect connection (i.e., marine water enters the cave through fissures and pores of the rock; Calderón‐Gutiérrez et al., 2018). Anthropogenic influences, such as groundwater pollution, water overexploitation, habitat destruction (i.e., quarrying of limestone), and alteration of adjacent ecosystems are of concern, and may lead to irreversible ecosystem change (Doehring & Butler, 1974; Iliffe, 2002; Mammola, Cardoso, et al., 2019). Meteorological events also appear to have an impact in the KSEs, with evidence of changes in temperature (Coutino et al., 2017; Kovacs et al., 2017), salinity (Coutino et al., 2017), water level (Collins et al., 2015; Kovacs et al., 2017), and oxygen (Brankovits et al., 2022) after tropical cyclones.

Both meteorological and anthropogenic impacts on KSEs remain poorly studied. The mortality event of 2015 highlighted the need to better understand the community resilience (i.e., capacity to recover to pre‐disturbances conditions). Therefore, the present study aimed to provide insights into precipitation impacts on temperature and how this may impact associated faunal community resilience via the analysis of in situ data loggers and long‐term biological surveys conducted before and after the 2015 mortality event (2012–2021). Since environmental disturbances by meteorological events are expected to be uncommon in underground environments, we hypothesized that stygobiont and stygophiles fauna would not be resilient to heavy precipitation events.

## 2 METHODS

**FIGURE 3:**
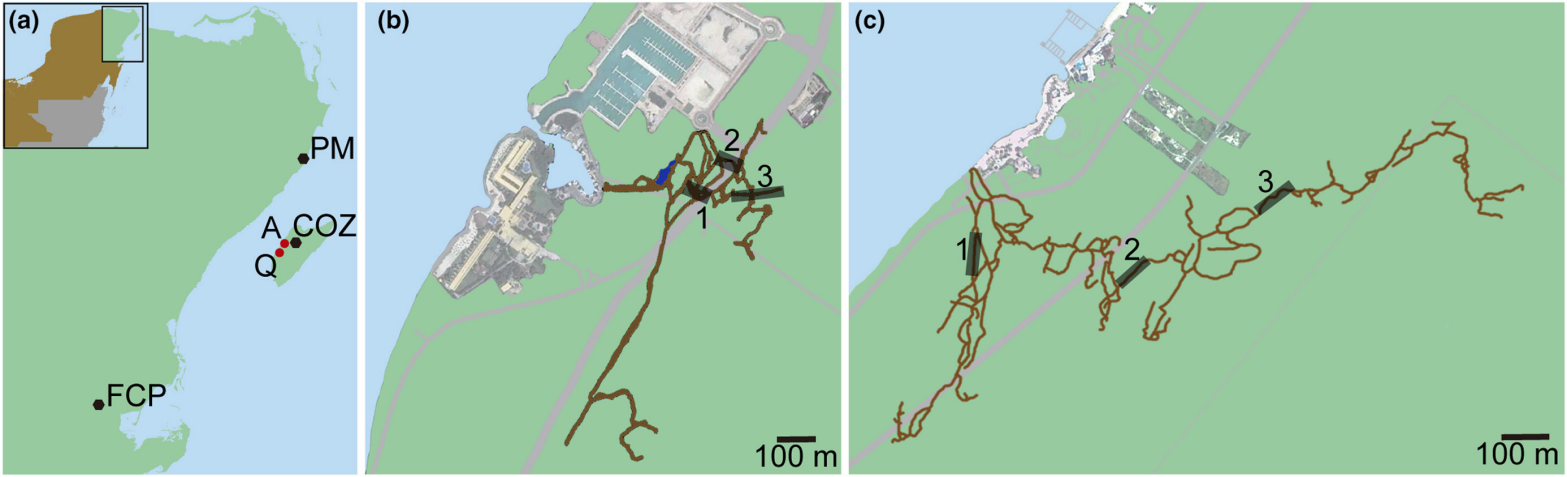
Map of the study area. (a) Caribbean Coast of the Yucatan Peninsula and Cozumel Island, showing the locations of the meteorological stations used to gather historical precipitation data (black dots; PM = Puerto Morelos, COZ = Cozumel, FCP = Felipe Carrillo Puerto). Location of caves systems (red dots): A = El Aerolito, Q = La Quebrada. Schematics of caves systems and biological survey sites (black boxes with numbers) on Cozumel Island: (b) El Aerolito and (c) La Quebrada. Modified from Calderón‐Gutiérrez et al. (2018).

This study focuses on the caves, El Aerolito and La Quebrada, Cozumel Island (Figures 1, 2, 3). Both caves are located at the center of the west coast of Cozumel Island, are directly connected to the ocean, shallow (average depth of 12 and 6 m, maximum of 27 and 9.7 m, on the caves El Aerolito and La Quebrada, respectively), and long (18 and 9.2 km, respectively). Considering the average depth, the distance between the halocline and the bottom of the cave is 5 m at El Aerolito, and 1.5 m at La Quebrada. They display a diverse community of stygobionts and marine species that includes annelids, cnidarians, chordates, crustaceans, echinoderms, mollusks, platyhelminths, and sponges (Calderón‐Gutiérrez et al., 2018; Yañez‐Mendoza et al., 2007).

### 2.1 Data collection of temperature and precipitation

In order to assess how the temperature was affected by precipitation, temperature data loggers (HOBO Pendant UA‐002‐64 Onset) that recorded information from before, during, and after the mortality event observed in El Aerolito in October 2015 were analyzed. Three sensors were installed at El Aerolito, at 5 m depth at the cave entrance, at 11 m depth at the end of site 3, and at 18 m depth at the start of site 2; another sensor was installed at La Quebrada, at 5 m depth at the start of site 1 (Figures 2 and 3). All the sensors were in the marine groundwater layer from January 2015 to July 2016 (Calderón‐Gutiérrez et al., 2018).

**FIGURE 4:**
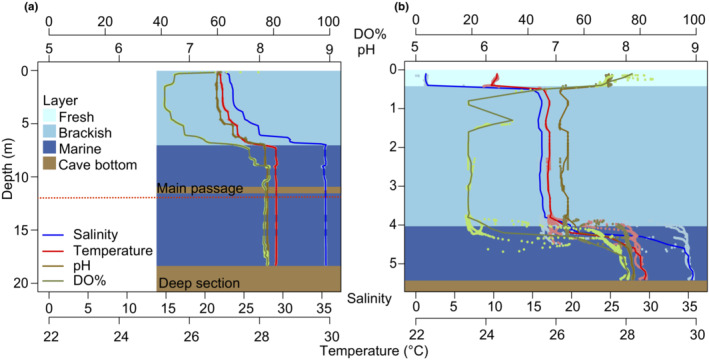
Hydrogeological profiles from November 2021 measuring depth, pH, dissolved oxygen (%), salinity, and temperature (dots = measurements; lines = average every 10 cm). Two water profiles were aligned to display a continuous single profile (see black shaded areas in Figure 2; profiles 1 at the entrance, profile 2 inside of the cave). (a) El Aerolito (profile 1 = 0–8.870 m, profile 2 = 8.997–18.258 m). Red dotted line indicates the depth of the halocline during the heavy precipitation event in 2015 at El Aerolito. (b) La Quebrada (profile 1 = 0–3.110 m, profile 2 = 0.821–5.401 m).

Precipitation data was obtained from the automatic meteorological stations from the Mexican National Meteorological System (SMN—CONAGUA, https://smn.conagua.gob.mx/es/observando‐el‐tiempo/estaciones‐meteorologicas‐automaticas‐ema‐s) from Cozumel and Felipe Carrillo Puerto, and the metereological station of the Marine and Limnology Sciences Institute (ICMyL—UNAM, https://sammo.icmyl.unam.mx/) from Puerto Morelos (Figure 3a). Meteorological data from Cozumel Island are available since May 5, 2013. Meteorological data from the Caribbean Coast of the Yucatan Peninsula are available at Felipe Carrillo Puerto since January 1, 1953, and at Puerto Morelos since September 14, 2017 (Figure 4g–i, Table 1). Felipe Carrillo Puerto, situated at 30 km from the coast, was included in this study to estimate the magnitude of the temperature variability due to precipitation since it is the meteorological station with the longest record available within the region of the study. The maximum accumulated precipitation over 4 days was measured at Felipe Carrillo Puerto, with 348.5 mm in June 1954.

**TABLE 1:** Historical meteorological data of precipitation accumulated over 4 days on the Caribbean Coast of the Yucatan Peninsula and Cozumel Island, and estimated maximum temperature decrease within the KSE in accordance with the generalized least squares (gls) model (Figure 5b–f).

Weather station	Data availability (days)	Days with accumulated precipitation (mm) over 4 days				Estimated maximum temperature decrease (°C)
		≥30	≥100	≥200	Maximum	
Cozumel Island	2078	282	46	2	227.08	0.89–1.86
Felipe Carrillo Puerto	24,094	3258	302	32	348.5	1.36–2.86
Puerto Morelos	707	61	11	0	160.9	0.63–1.32

Water profiles were measured in November 2021 at El Aerolito and La Quebrada with a multiparameter sonde YSI EXO1 with temperature, conductivity, pH, and dissolved oxygen sensors. All sensors were calibrated in accordance with the manufacturer. Two water profiles (at the entrance from the surface to the bottom and at the beginning of the deep passage inside of the cave from the ceiling to the bottom) were aligned to display a continuous profile (Figure 2).

### 2.2 Biological surveys

Specific density of conspicuous species (0.5–55 cm) was measured with 15 m^2^ (15 × 1 m) belt transects in triplicate by scientific cave divers. Biological surveys were performed at three sites per cave at El Aerolito and La Quebrada (Figures 2 and 3). Biological surveys at El Aerolito were carried out in January 2012, July 2012, January 2015, July 2018, January 2019, June 2019, and November 2021. It was possible to survey the three sites at El Aerolito in each campaign. Biological surveys at La Quebrada were carried out in January 2015, July 2018, July 2019, and November 2021. It was not possible to survey the three sites at La Quebrada in each campaign: Site 1 was surveyed in January 2015, July 2018, and November 2021; site 2 in January 2015, July 2018, and July 2019, and site 3 in January 2015, July 2018, July 2019, and November 2021. Surveys from 2012 to 2015 are published in Calderón‐Gutiérrez et al. (2018).

### 2.3 Analysis of the effects of precipitation on the temperature of the KSE

In order to analyze the effects of precipitation over KSEs in a period without extreme meteorological events (i.e., tropical cyclones), we compared water temperature with precipitation. First, linear regression of daily average of the temperature and daily standard variation of the temperature with precipitation, date, and site were performed to identify explanatory variables to temperature changes. Then, we used generalized additive mixed models (GAMM), allowing non‐linear relationships between the response variable and multiple explanatory variables by means of a smoothing function (Wood, 2004; Zuur et al., 2009). Two GAMM models were generated: (1) GAMM base model, to create a base model of the temperature without the influence of precipitation; and, (2) GAMM + precipitation model, to explain the effects of precipitation on the temperature of the KSE.

#### 2.3.1 GAMM base model

The GAMM base model was performed to evaluate the interannual variations of the temperature at KSEs. For this model, the average temperature per day was contrasted with the date using a Gaussian distribution. Penalized splines were used to have a smoother representation of the date (Wang et al., 2016; Wood, 2004). GAMM models with autocorrelation structure of order 1, precipitation with and without penalized splines as a second fixed variable, sites in the cave as random effects variables, and allowing different variances per site were tested. The best GAMM model was selected by comparison of the second‐order Akaike information criterion (AIC_c_; Burnham & Anderson, 2002), visually comparing the model results with the raw data, and performing diagnostics on the fitting procedure and results. The GAMM model was subsequently refined using different knots (i.e., location of joins on the spline curve), and the best model was selected as described before. These analyses were performed in R (R Core Team, 2021) with the mgcv 1.8‐38 (Wood, 2017) package for GAMM models, and MuMIn v1.43.17 (Bartoń, 2018) package for AIC_c_.

#### 2.3.2 GAMM + precipitation

The GAMM + precipitation model was performed to evaluate the impact of precipitation over the temperature at KSEs. For this model, the daily standard deviation of the temperature was compared with the precipitation from Cozumel (the closest meteorological station to the caves), considering the temporal lag at which temperature responds to precipitation. The temporal lag was estimated by contrasting the residuals of the GAMM base model with precipitation. GAMM models with and without penalized splines for accumulated precipitation, autocorrelation structure of order 1, sites in the cave as random effects variables, and allowing different variances per site were tested. The best GAMM + precipitation model was selected as described before. Accumulated precipitations over 30 mm in 4 days resulted in a drop of the temperature not explained by the GAMM base model.

#### 2.3.3 Impact of the precipitation on temperature

Linear regressions and generalized least squares (gls) were used to identify the impact of the precipitation on the temperature of the KSE. Linear regressions and gls of the residuals of the GAMM base model were performed with precipitation using a 4‐day lag, as identified in the GAMM model + precipitation. Variations of the models included using all the residuals, negative residuals, or residuals only under the lower confidence interval of the GAMM base model, all precipitation data, or accumulated precipitation of at least 30 mm, using sites in the cave or depth of the sensor as second variable, without and with interaction. Gls were also tested with site depths as an independent factor, and autocorrelation structure of order 1. The best model was selected by comparison of the *r* ^2^ and AIC_c_ (Burnham & Anderson, 2002). Then, gls with the same parameters as the best model were performed by site. Precipitation accumulated over 4 days was calculated from the historical data for the three meteorological stations, and the number of times when the precipitation was over 30 mm was identified. These analyses were performed in R (R Core Team, 2021) with the nlme v3.1‐153 (Pinheiro et al., 2021) package for gls, and MuMIn v1.43.17 (Bartoń, 2018) package for AIC_c_.

### 2.4 Analysis of the community resilience to precipitation impacts

The community and species‐specific response to the mortality event observed in October 2015 at El Aerolito was done by comparing each biological surveys of macrofauna (Figures 2 and 3; Calderón‐Gutiérrez et al., 2018). The community structure from each biological survey was compared by analyzing the species richness (count of the number of species) and changes in the density of organisms, for all the sites, independently of the cave, through a non‐metric multidimensional scaling analysis (nMDS). Temporal changes in beta diversity (Δ*β*) were quantified with an extirpation‐colonization analysis (Tatsumi et al., 2021, 2022).

In order to select the fittest nMDS, 19 dissimilarity indices were tested (Binomial, Bray Curtis, Cao, Canberra, Chao, Chisq, Chord, Clark, Euclidean, Gower, modified Gower, Horn, Jaccard, Kulczynski, Mahalanobis, Manhattan, Morisita, Mountford and Raup). The nMDS with a convergent solution and lower stress was selected. The goodness of fit was tested by a Shepard diagram (Legendre & Legendre, 2012).

Extirpation‐colonization analyses performed pairwise comparisons of specific densities at two different times and then partition the total Δ*β* changes due to extirpation (i.e., local extinction) and colonization (Tatsumi et al., 2021, 2022). The extirpation‐colonization analyses were realized by cave, comparing (1) all the fauna recorded in the biological surveys, (2) Stygobionts, and (3) Stygophiles in R (R Core Team, 2021) with the R package ECOPART v0.2.0 (Tatsumi et al., 2022) using the Whittaker index. Stygophiles and stygoxenes were grouped together as stygophiles since current natural history information does not allow their differentiation for most species, while species with stable populations are expected to be stygophiles rather than stygoxenes. One sample *t‐*test against 0 was used to identify substantial changes in *β* (Tatsumi et al., 2021); an *α* of 0.1 was used since the heterogenicity distribution of the fauna in both caves (Calderón‐Gutiérrez et al., 2018) was expected to disguise temporal variability. Because not all the sites were surveyed during each campaign at La Quebrada, extirpation‐colonization analyses from this cave were restricted to sites 2 and 3, and the years 2015, 2018, and 2019. Priority to these sites and times was used since they corresponded with the time of the mortality event observed at El Aerolito. Changes in the density of each species were analyzed with a Kruskal–Wallis (data did not have normality nor homoscedasticity) and by graphical comparison with box plots. Due to the low population sizes, an *α* = 0.1 was used on the Kruskal–Wallis test.

These analyses were performed in R (R Core Team, 2021) with the vegan v2.5‐7 package (Oksanen et al., 2014) for nMDS and the ECOPART v0.2.0 (Tatsumi et al., 2022) package for the extirpation‐colonization analysis.

### 2.5 A priori testing and data handling

A priori assumptions were tested with the Shapiro–Wilk (SW) test for normality, Breusch–Pagan (B‐P) test for heteroscedasticity of the regression analysis, and Barlett test for heteroscedasticity of the species densities (Zar, 2010). Data handling was performed in R (R Core Team, 2021) with the dplyr v1.0.7 (Wickham et al., 2022), ggplot2 v3.3.5 (Wickham, 2016), lubridate v1.8.0 (Grolemund & Wickham, 2011), readbulk v1.1.3 (Kieslich, 2016), and tidyr v1.1.4 (Wickham & Girlich, 2022) packages.

## 3 RESULTS

Depth of the halocline adjacent to the marine groundwater layer at El Aerolito and La Quebrada was 7 and 4.5 m, respectively, during a day without precipitation. All measured parameters in the water profile changed at the halocline, not only salinity (Figure 4). Two water layers were detected at El Aerolito (Figure 4a), and three water layers, from fresh to marine groundwater, were detected at La Quebrada (Figure 4b).

### 3.1 Effects of precipitation on the KSE

Linear regression of temperature with precipitation, date, and site showed a relation of date with temperature (*r* ^2^ = .20, *n* = 2136). No other relation was detected (Figure S1). Linear regressions of the standard deviation of the temperature evidenced a positive relation with precipitation (*r* ^2^ = .40, *n* = 2136) and a negative relation with sites (*r* ^2^ = .26, *n* = 2136; Figure S1).

**FIGURE 5:**
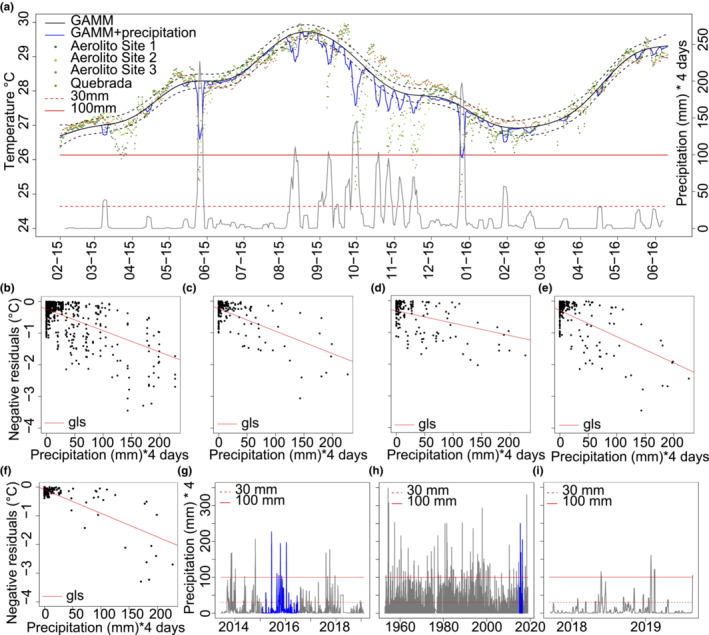
Effects of precipitation on the water temperature of the KSE of Cozumel Island. (a) GAMM models (base model *r* ^2^ = .74, *k*' = 14, edf = 11.9, *p* < .05, with precipitation *r* ^2^ = .81, *k*' = 14, edf = 11.9, *p* < .05), with confidence interval, of the marine groundwater temperature compared with precipitation. Gls of the GAMM model residuals versus accumulated precipitation over 4 days from: (b) All sites, (c) El Aerolito Site 1 (depth = 5 m), (d) El Aerolito Site 2 (depth = 18 m), (e) El Aerolito Site 3 (depth = 11 m), (f) La Quebrada (depth = 5 m), corrected for autocorrelation. The temperature decreases 0.0068, 0.0039, 0.0079, and 0.0082°C, respectively, per mm of precipitation accumulated over 4 days (*p* < .05). Accumulated precipitation over 4 days in the Mexican Caribbean at (g) Felipe Carrillo Puerto, (h) Cozumel, and (i) Puerto Morelos, data highlighted in blue corresponds to the dates in Figure (a).

The GAMM base model of the temperature (*r* ^2^ = .74, *k*' = 14, edf = 11.9, *p* < .05) included the date with splines with 15 knots and an autocorrelation structure of first order following a Gaussian distribution (Figure 5a). The obtained model was able to subtract the daily variation of measured temperature, showing temperature seasonality, with the highest temperature in August and the lowest in February. The GAMM base model (Figure 5a; Figure S1) has neither homoscedasticity (B‐P < 0.05) nor normality (SW < 0.05, *n* = 2136).

Comparison of the residuals of the GAMM base model with precipitation suggested a lag of 3–4 days, meaning that temperature changes depended on the accumulated precipitation over 3–4 days. A second GAMM model, here‐after called GAMM + precipitation (Figure 5a; Figure S1), indicated a direct relation of the fitted values below the confidence interval of the GAMM base model per accumulated precipitation above 30 mm over 4 days (*r* ^2^ = .81, *k*' = 14, edf = 11.9, *p* < .05). The precipitation model improved homoscedasticity (B‐P > 0.05), but did not have normality (SW < 0.05, *n* = 1869).

Linear regression of the negative residuals of the GAMM base model (Figure S1), depth, accumulated precipitation over 4 days, accumulated precipitation above 30 mm, and site resulted only in a negative relation of the precipitation (*r* ^2^ = −.68, *n* = 1869), and with the accumulated precipitation above 30 mm (*r* ^2^ = −.51, *n* = 1869). It also showed strong autocorrelation. The best model to explain the effects of precipitation in the negative residuals of the GAMM base model was generalized least squares fit (gls) with an autocorrelation structure of first order (Figure 5b–f), following a Gaussian distribution (*p* < .05). In accordance with this model, the temperature decreased 0.0068°C per mm of precipitation accumulated over 4 days. The model estimated a decrease in temperature of 0.0079–0.0082°C/mm of precipitation (*p* < .05) within La Quebrada, El Aerolito Site 1, and Site 3 (Figure 5c,e,f). A halocline is present within each of these sites, and the floor of these sites has a maximum depth of 11 m (Figures 1, 2 and 4). The estimated decrease in temperature at El Aerolito Site 2 (depth = 18 m; halocline absent) was 0.0039°C (*p* < .05; Figures 2 and 5d). However, the GAMM + precipitation model underestimated the magnitude. For example, the temperature on October 21, 2015, was 2.5–4.7°C lower than on October 17, 2015 (El Aerolito Site 2 and La Quebrada, respectively), this temperature drop was almost of the same magnitude than the interannual temperature variability of 5.32°C. While the GAMM + precipitation model infers a temperature drop of 0.6–1.2°C, this is on average 28% of the observed change. The comparison of these results with the historical meteorological data of precipitation within the region (Table 1) shows that underwater temperature potentially decreased up to 2.37°C due to precipitation and up to 8.46°C considering the magnitude of the model underestimation, which is half to 1.5 times the interannual temperature variability (Figure 5).

### 3.2 Community resilience to heavy precipitation events

**FIGURE 6:**
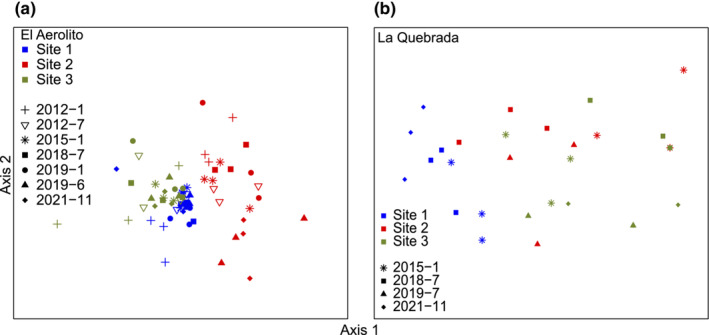
nMDS with the spatial and temporal distribution from (a) El Aerolito (stress = 0.186, best distance = Euclidean, non‐metric fit *r* ^2^ = .965) and (b) La Quebrada (stress = 0.0974, best distance = Mountford, non‐metric fit *r* ^2^ = .991).

Macrofaunal biological surveys of El Aerolito and La Quebrada in 2012 and 2015 (Calderón‐Gutiérrez et al., 2018), as well as 2018, 2019, and 2021 (this study) allowed the comparison of the community structure and species‐specific changes before and after the mortality event. Species richness, number of phyla, and densities fluctuated throughout the study (Table S1); however, significant change was not observed (Figure S2). A total of 63 species were recorded at El Aerolito, ranging from 18 to 32 species (January and July 2012, respectively), and a total of 26 species recorded at La Quebrada, ranging from 8 to 15 species (July 2019, November 2021, respectively). The community from both caves displayed a heterogenic distribution, with each site forming a group on both axes of the nMDS analysis (Figure 6). The samples from El Aerolito Site 2, the deepest site (18 m), and the only one without access to the halocline, clustered together, and had the least number (i.e., 28) of species (Figure 6a; Table S1, Figure S3). The samples from La Quebrada Site 1, the closest site to the coastline, clustered together. Site 1 is the area with most of the species of Porifera recorded. Sites 2 and 3 from La Quebrada are distinguished by axis 2 of the nMDS (Figure 6b; Table S1, Figure S4).

**FIGURE 7:**
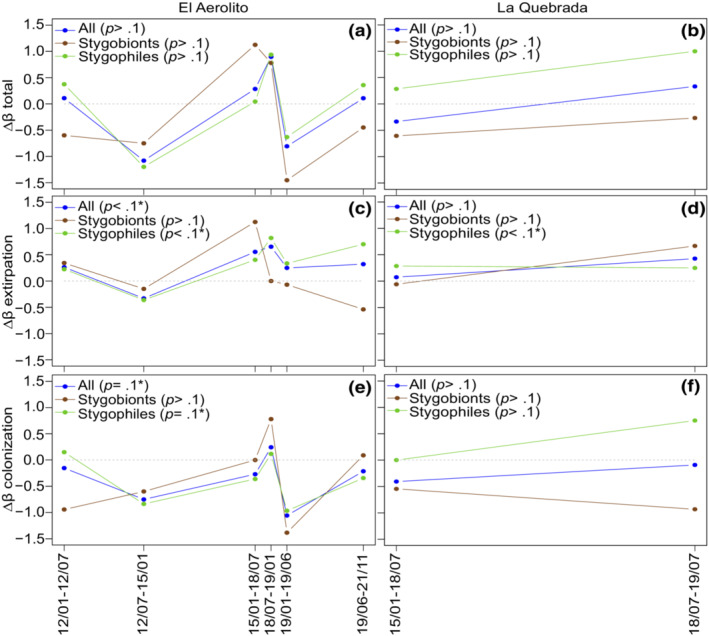
Temporal changes in beta diversity (Δ*β*) between pairs of biological surveys at El Aerolito (a, c, e) and La Querbrada (b, d, f). The total change in Δ*β* is divided into extirpation and colonization resultant changes. The analyses were performed for all species in the biological surveys, and by its ecological classification (Stygobiont and Stygophiles). *p*‐Values and *t*‐tests indicate statistical differences (statistical differences indicated with *) with 0, which would indicate no significant changes in beta diversity.

Effects of the precipitation event (and mortality event) were evaluated by analyzing the changes in beta diversity. No net changes (Δ*β* total) were detected in the overall community, neither when decomposing the community into stygobionts and stygophiles at El Aerolito nor La Quebrada (Figure 7a,b). Partitioning Δ*β* into its extirpation and colonization components revealed significant changes at El Aerolito for the overall community and stygophiles, while stygobionts did not have significant changes (Figure 7c,e). At La Quebrada, the only significant change in Δ*β* was due to the extirpation of stygophiles (Figure 7d,f).

Specific densities (Table S1, Figures S3 and S4) show the following trends: (i) Colonization. For example, the stygophile/stygoxene sponge *Clathrina hondurensis* (El Aerolito Site 2) was not present prior to the mortality event. (ii) Density increases in opportunistic species. For example, the stygobiont shrimp *Procaris mexicana* had very low densities before the mortality event (La Quebrada Site 3). These two types of species were recorded for the first time in 2018 and showed significant increases in density post‐mortality event. (iii) Extirpations or density decreases of impacted species. For examples, the stygophile sponge *Geodia neptuni* (El Aerolito Site 1) showed a density drop from ~40 org/100 m^2^ (pre‐mortality) to fluctuations (<30 org/100 m^2^) in 2016–2019, and then not recorded in 2021, while the stygophile ascidian *Didemnum* sp. (La Quebrada Site 3) significantly decreased from >70 org/100 m^2^ pre‐mortality to <10 org/100 m^2^ in 2018–2021. (iv) Constant density of resilient and resistant species. For example, densities of the stygobiont ophiuroid *Ophionereis commutabilis* (El Aerolito Site 3), and the stygobiont isopod *Metacirolana mayana* (Quebrada Site 3), were not significantly different. (v) Stygoxenes and stygophiles significant density changes compared with stygobionts. (vi) Higher density impacts occurred within sites closest to the coastline (site 1), displaying significant differences at the species level. Overall, 66% and 55% of the species at La Quebrada and El Aerolito, respectively, exhibited density change (Table S1, Figures S3 and S4).

Impacts of the mortality event on some stygobiont species were not possible to evaluate due to their naturally low population sizes. For example, the endemic sea star *Copidaster cavernicola,* only known from El Aerolito, was recorded three times in biological surveys: one each in January 2012 and July 2012 (site 3) and one in July 2018 (site 1). In November 2021, only four *C. cavernicola* individuals were observed after 32 h of effort (number of divers × length of the dive), at least half of that time was in areas where it had previously been observed. In the case of the remipede *Xibalbanus cozumelensis* (Figure 1i), small numbers have been observed in a chamber of the cave further inland than La Quebrada Site 3. Two individuals of *Xibalbanus* sp. have been observed at El Aerolito Site 1 in July 2018, and one further inland than site 3 at a depth of 27 m in November 2021. Observations of *Xibalbanus* were outside the biological survey transects.

## 4 DISCUSSION

This study provides insights into the effects of meteorological events on groundwater temperature within a KSE, with changes in groundwater temperature of half the magnitude than the interannual variation. Previous reports of changes in groundwater due to meteorological events have been related to tropical cyclones (Brankovits et al., 2022; Collins et al., 2015; Coutino et al., 2017; Kovacs et al., 2017). Evidence from the GAMM + precipitation shows that the groundwater is not only affected by extreme meteorological events. Results of the gls model quantified the temperature decrease by accumulated precipitation. However, the GAMM + precipitation model does not reflect the magnitude of the temperature drops due to precipitation, which could require data from different locations and different depths to be adjusted. Furthermore, temperature drops detected in June 13–14, 2015, and January 13–15, 2016, and the lack of previous observations of displacement of the halocline, suggest that the displacement of the halocline depends not only on the magnitude but also the duration of the events. For example, the event in October was the result of 8 days of heavy precipitation, while the other two precipitations events during this study of a similar magnitude had a duration of 2–3 days (Figure 5a). Meteorological historical data from the region show that underwater temperature potentially decreased up to 1.5 times the interannual temperature variability due to heavy precipitation. Displacement of the halocline is mainly due to mixing of the adjacent water masses, thus not only affecting salinity but other parameters, such as temperature (Figure 4; Smith et al., 2008). Accordingly, temperature changes not explained by the temporal variation are a good proxy to evaluate the impacts of meteorological events. Models of environmental parameters and their response to different events, both natural and anthropogenic, along with physiological information of the fauna on their tolerance thresholds would allow mortality event predictions (Castaño‐Sánchez et al., 2020; Weeks et al., 2008).

Water level and salinity changes have been observed to have a greater influence within caves near the coast, which could be due to the groundwater drainage moving the water from the upper meteoric water mass toward the coast (Beddows et al., 2007; Collins et al., 2015; Kovacs et al., 2017; Smith et al., 2008; Vera et al., 2012). Accordingly, communities inhabiting shallow caves, caves closest to the coastline, and/or sections where the halocline is near the cave bottom are more vulnerable to experiencing changes in higher frequency and magnitude. For example, due to the morphology of the cave, in El Aerolito the halocline to cave bottom depth at site 3 is on average 1 m deeper than at site 1; thus, a smaller event could impact site 1 but not site 3 (both sites have a similar species composition (Calderón‐Gutiérrez et al., 2018)). This could explain why more species at site 1 experienced significant changes during the period of this study (Table S1). Changes in species densities were also detected at deeper sites lacking direct access to the halocline where no changes were observed in October 2015 (El Aerolito Site 2, 18 m). We hypothesize that these changes were caused by direct and indirect mortality. Direct mortality (i.e., changes directly affecting fauna) could be a consequence of the drastic osmotic shock and temperature change brought in by the event (the day before when it was not possible to dive due to meteorological conditions). Available information of the variability of the environmental parameters is limited to temperature and water level, and information about the changes due to disturbances by severe weather is scarce (Brankovits et al., 2022; Collins et al., 2015; Coutino et al., 2017; Kovacs et al., 2017; Mejía‐Ortíz et al., 2020). Currently, no studies have been performed on the physiological tolerances of stygobiont species, or relatives at family level, present in Cozumel Island (Ballou et al., 2022). Indirect mortality (i.e., changes that provoke the alteration of other parameters affecting the fauna) could mainly be due to changes in the organic matter entering deeper sites as a consequence of alterations of the community in adjacent sections of the cave (Ban et al., 2008). Subtle changes in organic matter composition and concentration cause an increase in heterotrophic microbes leading to oxygen depletion (Brankovits et al., 2021; Cresswell & van Hengstum, 2021), thus affecting food and oxygen availability.

Changes in the community structure were detected in this study by decoupling the Δ*β* into colonization and extirpation events (Figure 7), otherwise disguised by the combination of both processes (Tatsumi et al., 2021). *β* diversity changes were the result of variations of stygophile/stygoxene, contrary to the hypothesis that stygobionts would be as vulnerable. Species within the three ecological categories inhabiting both caves are composed of both benthic species (i.e., marine sponges) and mobile species (i.e., crustaceans and echinoderms; Calderón‐Gutiérrez et al., 2018); therefore, the resilience resistance displayed by stygobionts but not by stygophiles/stygoxenes cannot be attributed to the capacity of the organisms to move at the time of the mortality event. At La Quebrada, the only change in *β* diversity detected was on extirpation of stygophiles, but not in the overall community. These observations concur with stygophiles being more susceptible to environmental changes. The two main differences between El Aerolito and La Quebrada are that the latter has a lower species richness and a halocline closer to the bottom (Calderón‐Gutiérrez et al., 2018). This may suggest that events displacing the halocline below the average bottom depth due to precipitation are more frequent, and thus, organisms inhabiting at La Quebrada are more resilient to these events, also explaining the lower species richness (i. e., four times lower) in comparison with El Aerolito. KSEs are dynamic ecosystems from the optics of geological times and sea level changes (Bishop et al., 2015; Brankovits et al., 2018; Rohling et al., 2021) that expand and contract through time, while it is unknown how the fauna moves with these changes. Adaptations of stygobiont species (i.e., physiological: tolerance to changes, ethological: vertical migrations before or at the beginning of similar events) to the dynamic nature of the KSEs could explain why they were resilient to this mortality event. Disturbances on the stygophiles, and especially stygoxenes, suggest that these species are already under stressful conditions at KSEs by living in a cave environment, making them more susceptible to disturbances (Moldovan et al., 2018). However, changes in this ecosystem due to sea level are usually through generations, and not stochastic or short‐term events, as such, it should not be assumed that the stygobiont fauna will be resilient to future similar events, especially if the frequency and magnitude increases, as many model predicts (Emanuel, 2017; IPCC, 2022; Siegert et al., 2020). Furthermore, relationships between stygobionts, stygophiles, and stygoxenes have not been studied on KSEs. Changes in stygophiles and stygoxenes populations and communities have the potential to alter the stygobionts, including micro‐endemic species, due to competition, predation, and food availability.

Heavy rainfall has been recognized to cause severe mortality in other aquatic communities, such as coral reefs (Huang et al., 2014), seagrasses (Webster et al., 2021), and even microbial communities (Woods et al., 2022), where each community displays different degrees of resilience, from weeks (microbes) to years (seagrass and coral reefs). These events are expected to become more frequent and with a higher magnitude due to climate change (Hoegh‐Guldberg & Bruno, 1979; IPCC, 2022; Siegert et al., 2020), while the impact of climate change to KSEs remains unknown (Mammola, Cardoso, et al., 2019; Mammola, Piano, et al., 2019). The mortality events could also increase in frequency and magnitude, representing a threat, especially for species with naturally low population sizes and small distribution ranges, such as stygobionts (Mammola, Cardoso, et al., 2019). A better understanding of ecosystem thresholds of disturbance and resilience allows the identification of proxies to recognize an ecosystem under pressure, and to implement mitigation and restoration plans (Thompson et al., 2020). We recognize the complications of conducting research and ecological monitoring of populations with low densities, such as the sea star *Copidaster cavernicola* and the remipede *Xibalbanus cozumelensis*, thus future research should include species‐specific projects to understand the vulnerability of endemic species (Rayment et al., 2011). Thus, preference should be given to non‐extractive techniques, such as visual methods and environmental DNA (eDNA; Benítez et al., 2019; Calderón‐Gutiérrez et al., 2018; Saccò et al., 2022). The latter has the advantage of detecting the presence of a species without direct observation, however, the semi‐isolated characteristics of the KSE minimizes one of the major caveats of eDNA, that is, DNA origin uncertainties (Saccò et al., 2022).

In conclusion, this study found that temperature from aquatic caves with direct connection to the ocean are impacted by rainfall, even at deeper sites within the cave. Future projects should evaluate the effects of precipitation on other environmental parameters including salinity, depth of the halocline(s), dissolved oxygen, and organic matter. While changes in the species presence and species density of non‐stygobiont marine species were detected, the KSE communities were resilient to the mortality event caused by heavy precipitation in 2015. Future exploration of the physiology of stygobionts, the ecological relationships between stygobionts species and marine inhabitants (i.e., stygoxenes and stygophiles), and the interactions with adjacent ecosystems will expand our understanding of resilience thresholds of aquatic cave communities. An evaluation of the precipitation impact on temperature to subterranean estuaries, in other regions, with direct connection to the ocean should be performed to test whether the values here reported are applicable to other areas.

## AUTHOR CONTRIBUTIONS

**Fernando Calderón Gutiérrez:** Conceptualization (equal); data curation (equal); formal analysis (equal); funding acquisition (equal); investigation (equal); methodology (equal); visualization (equal); writing – original draft (equal); writing – review and editing (equal). **Thomas M. Iliffe:** Conceptualization (equal); funding acquisition (equal); methodology (equal); supervision (equal); writing – review and editing (equal). **Elizabeth Borda:** Conceptualization (equal); funding acquisition (equal); investigation (equal); methodology (equal); supervision (equal); writing – review and editing (equal). **German Yañez:** Conceptualization (equal); investigation (equal); methodology (equal); resources (equal); writing – review and editing (equal). **Jessica Labonté:** Conceptualization (equal); funding acquisition (equal); investigation (equal); methodology (equal); supervision (equal); validation (equal); writing – review and editing (equal).

## FUNDING INFORMATION

This work was supported by the Texas A&M University‐San Antonio (startup funds), Consejo Nacional de Ciencia y Tecnología (CONACYT—grant No. 472273), CONACYT‐Texas A&M University (Grant No. 2018‐044‐1), and The Mohamed bin Zayed Species Conservation Fund (Grant No. 200523736).

## CONFLICT OF INTEREST STATEMENT

The authors of this manuscript have nothing to disclose.

### OPEN RESEARCH BADGES

This article has earned Open Data, Open Materials and Preregistered Research Design badges. Data, materials and the preregistered design and analysis plan are available at: https://doi.org/10.5281/zenodo.7523431.

## Supporting information


Figure S1

Figure S2

Figure S3

Figure S4

Table S1

## Data Availability

The data and code that support the findings of this study are openly available in the Zenodo repository at: https://doi.org/10.5281/zenodo.7523431 (Calderón‐Gutierrez et al., 2023).
