# Supplementary material for: Response and resilience of karst subterranean estuary communities to precipitation impacts
Source: Ecol Evol. 2023 Aug 14;13(8):e10415. doi: 10.1002/ece3.10415 (PMC10425610; doi:10.1002/ece3.10415)

Supplementary figure 1.- Linnear regressions for data exploration and diagnostic plots for GAMM models.

Linear regressions. Font size of r² values are proportional to its value.

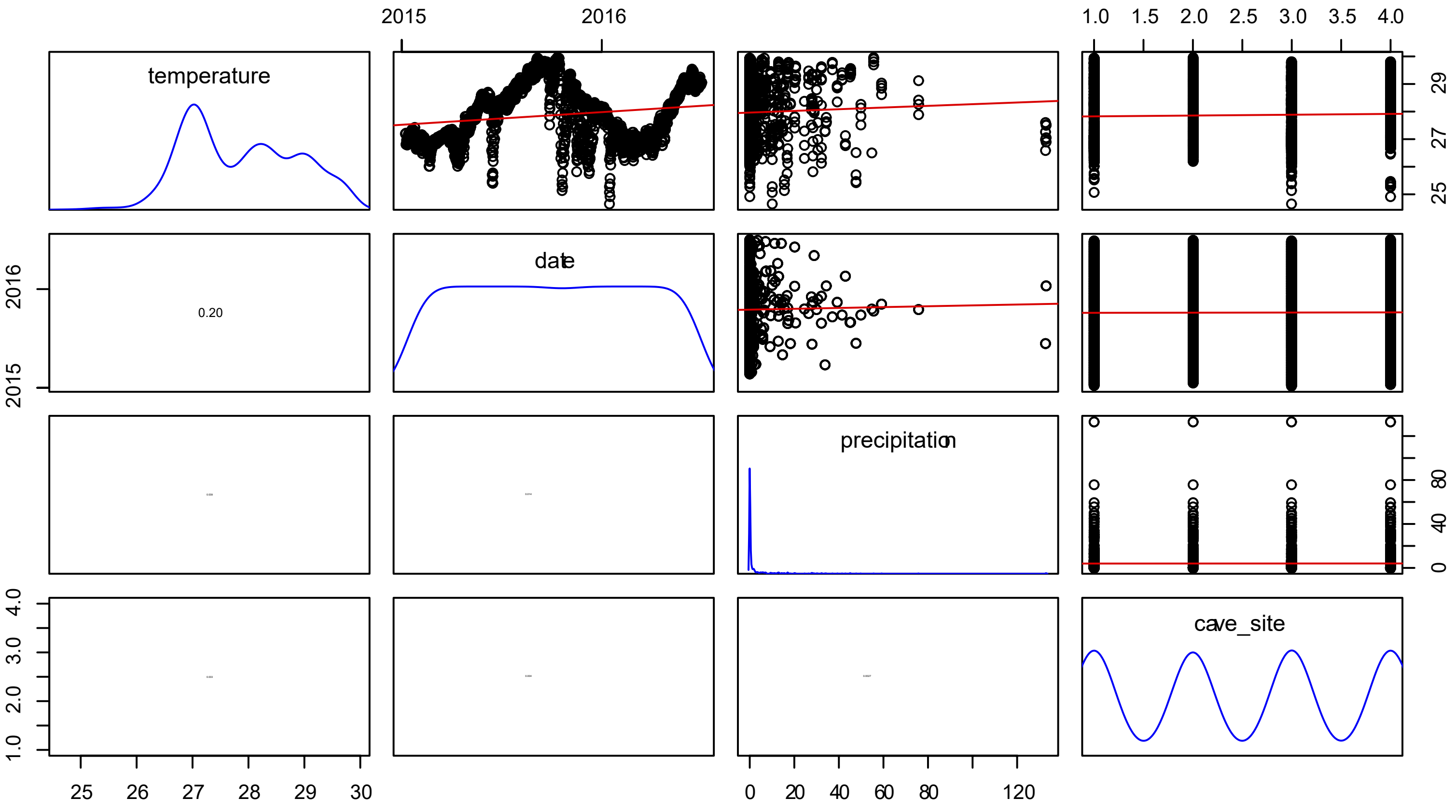

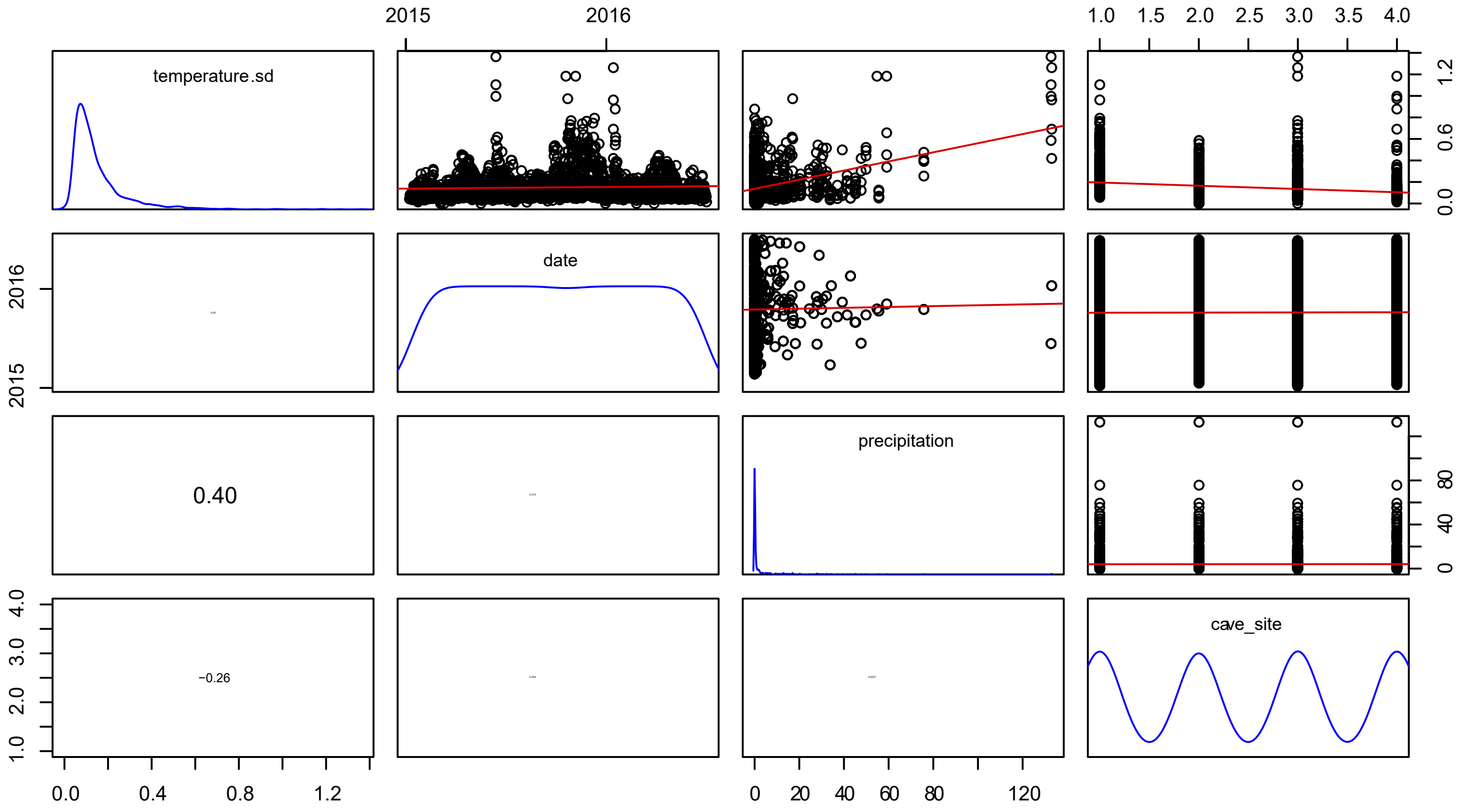

Diagnostic plots from GAMM models

GAMM base model

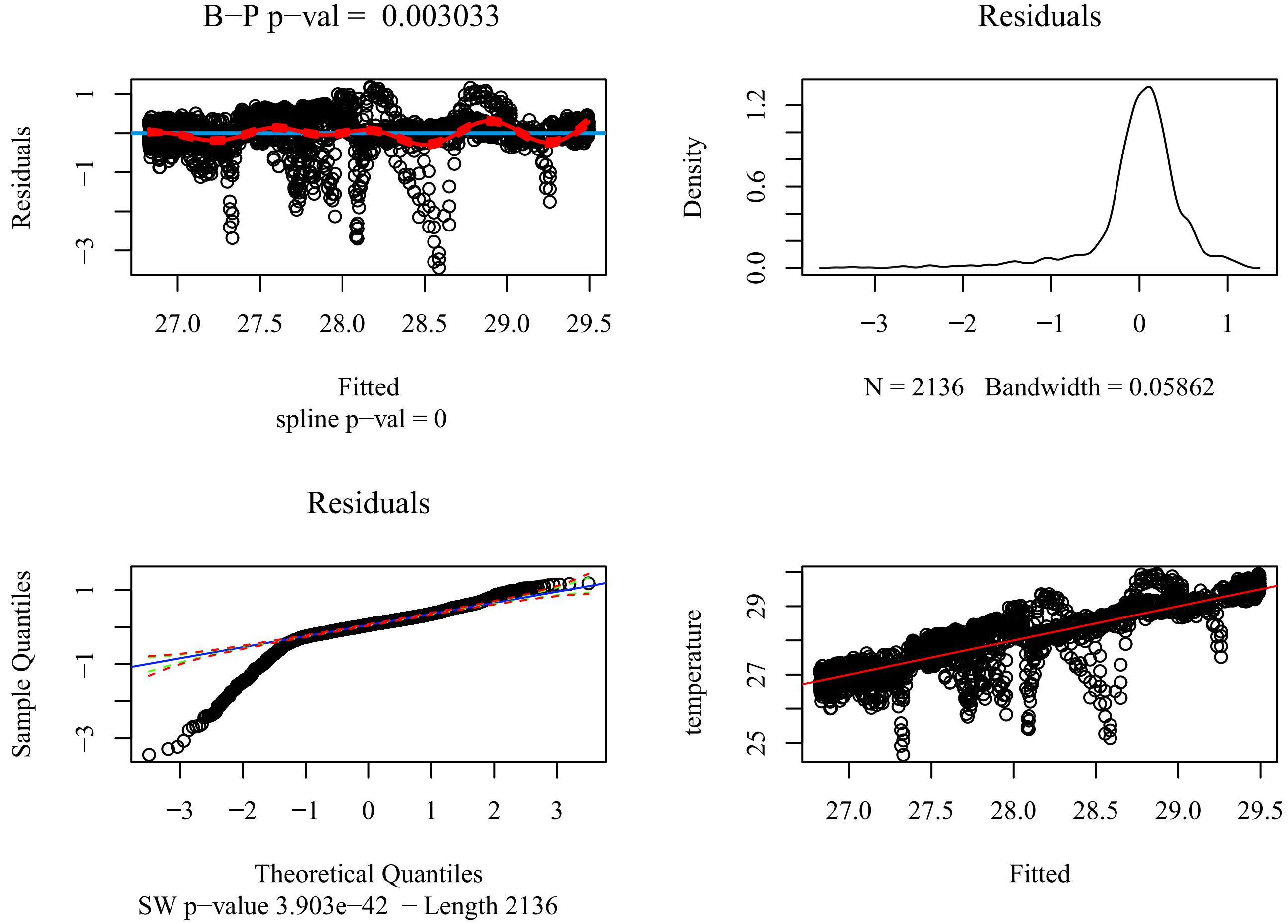

GAMM + precipitation model

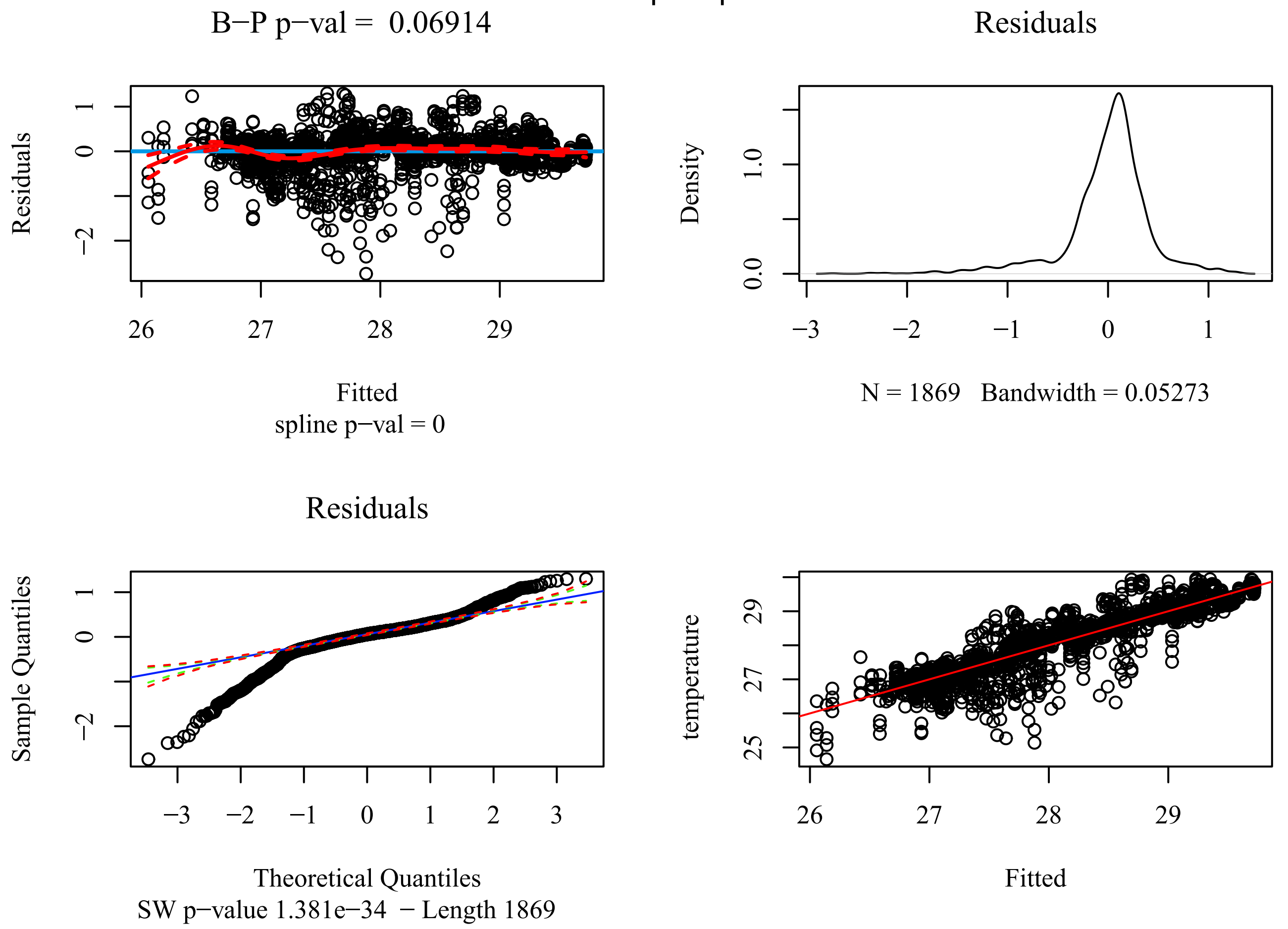

Supplement: Supplementary file 1 — Figure S1 [file ECE3-13-e10415-s003.pdf]
