# Supplementary material for: Response and resilience of karst subterranean estuary communities to precipitation impacts
Source: Ecol Evol. 2023 Aug 14;13(8):e10415. doi: 10.1002/ece3.10415 (PMC10425610; doi:10.1002/ece3.10415)

Supplementary figure 2.- Species richness and density through the study at a, c) El Aerolito and b, d) La Quebrada. Only positive standard deviation is displayed

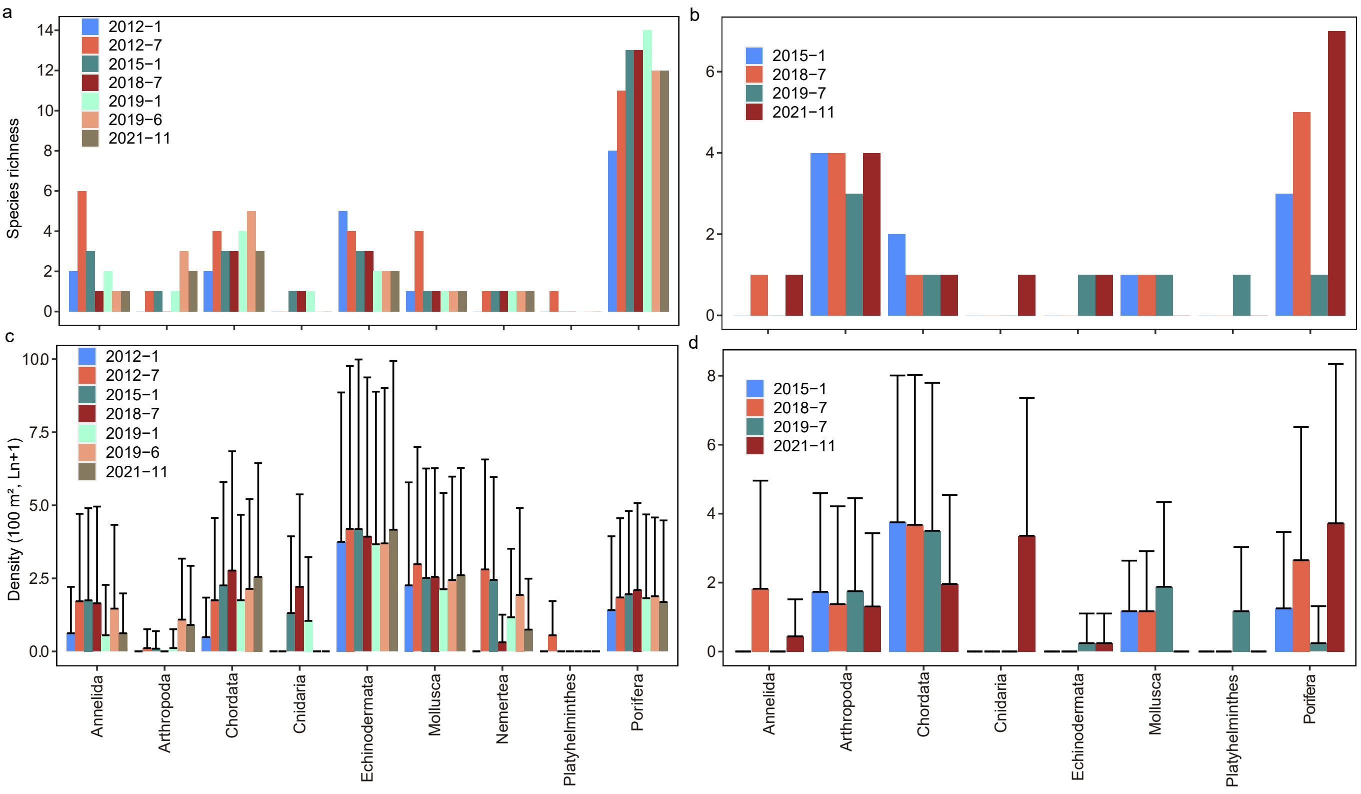

Supplement: Supplementary file 2 — Figure S2 [file ECE3-13-e10415-s004.pdf]
