# Supplementary material for: Response and resilience of karst subterranean estuary communities to precipitation impacts
Source: Ecol Evol. 2023 Aug 14;13(8):e10415. doi: 10.1002/ece3.10415 (PMC10425610; doi:10.1002/ece3.10415)
Supplement: Supplementary file 3 — Figure S3 [file ECE3-13-e10415-s005.pdf]

Supplementary figure 3.- Densities of each species recorded in El Aerolito during the ecological census. (* Stygobiont)

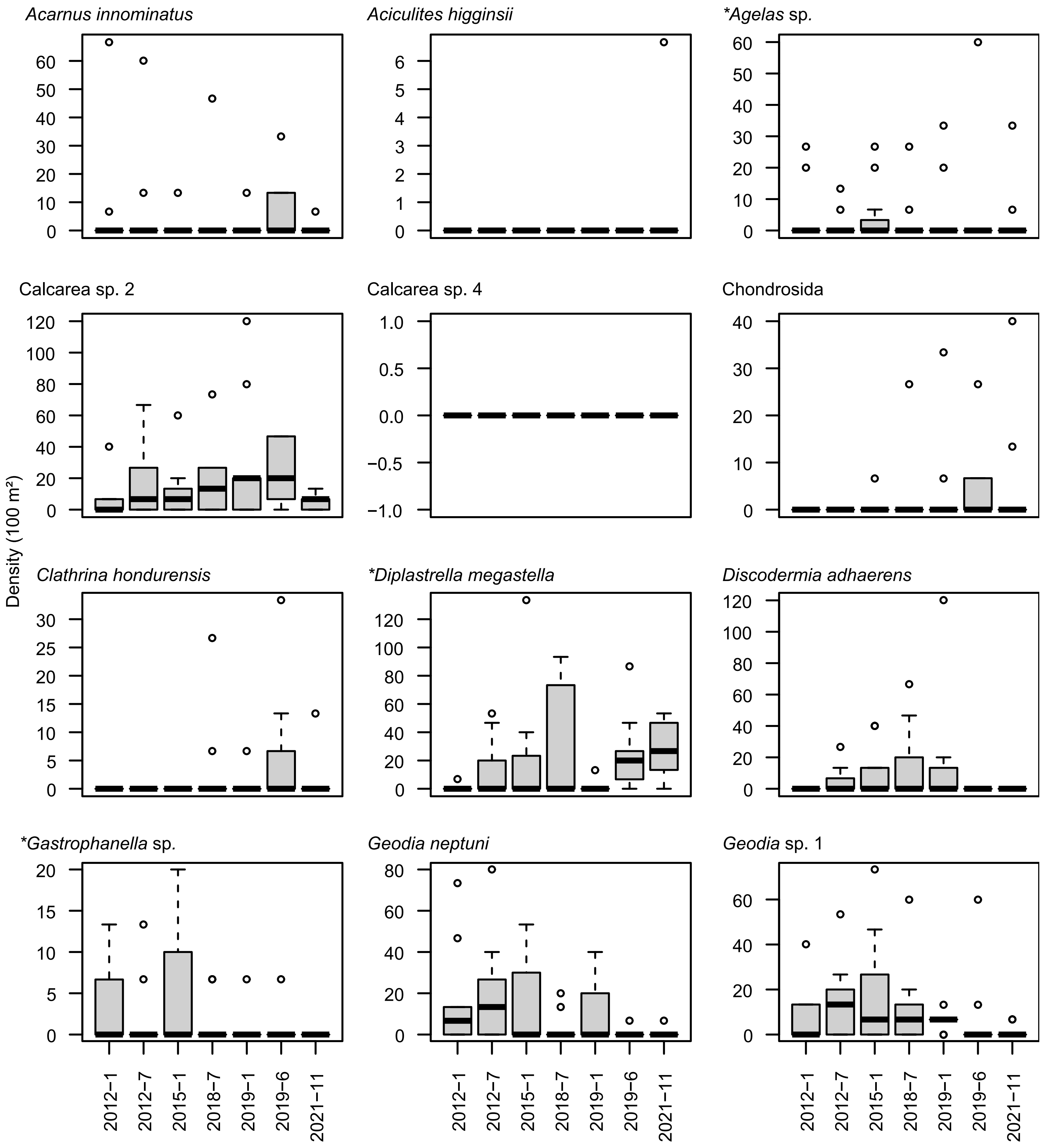

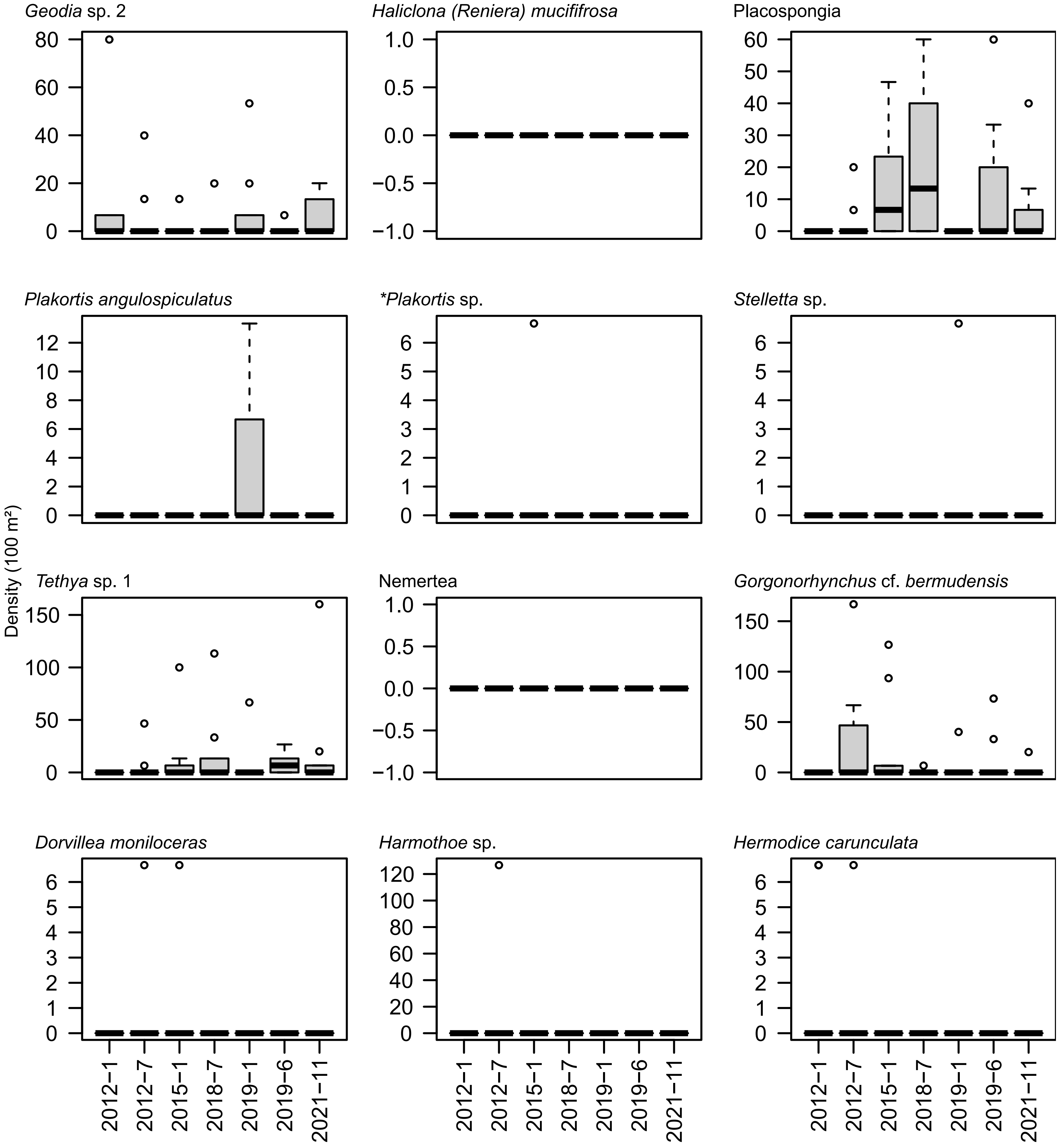

Geodia sp. 2
Haliclona (Reniera) mucififrosa
Placospongia
Plakortis angulospiculatus
**Plakortis* sp.
Stelletta sp.
Tethya sp. 1
Nemertea
Gorgonorhynchus cf. *bermudensis*
Dorvillea moniloceras
Harmothoe sp.
Hermodice carunculata
Density (100 m²)
2012–1
2012–7
2015–1
2018–7
2019–1
2019–6
2021–11

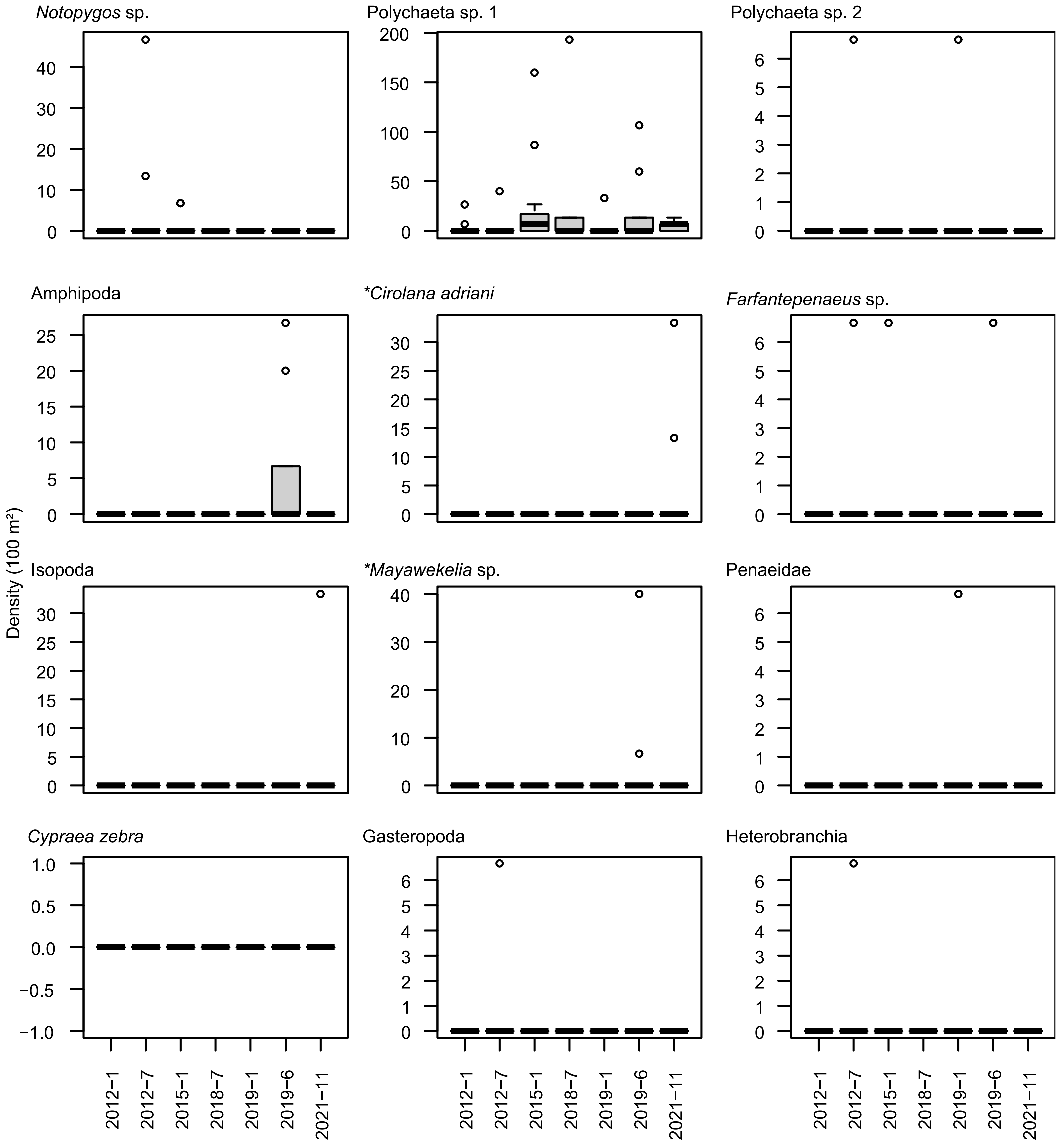

Notopygos sp.
Polychaeta sp. 1
Polychaeta sp. 2
Amphipoda
*Cirolana adriani
Farfantepenaeus sp.
Isopoda
*Mayawekelia sp.
Penaeidae
Cypraea zebra
Gasteropoda
Heterobranchia
Density (100 m²)
2012–1
2012–7
2015–1
2018–7
2019–1
2019–6
2021–11

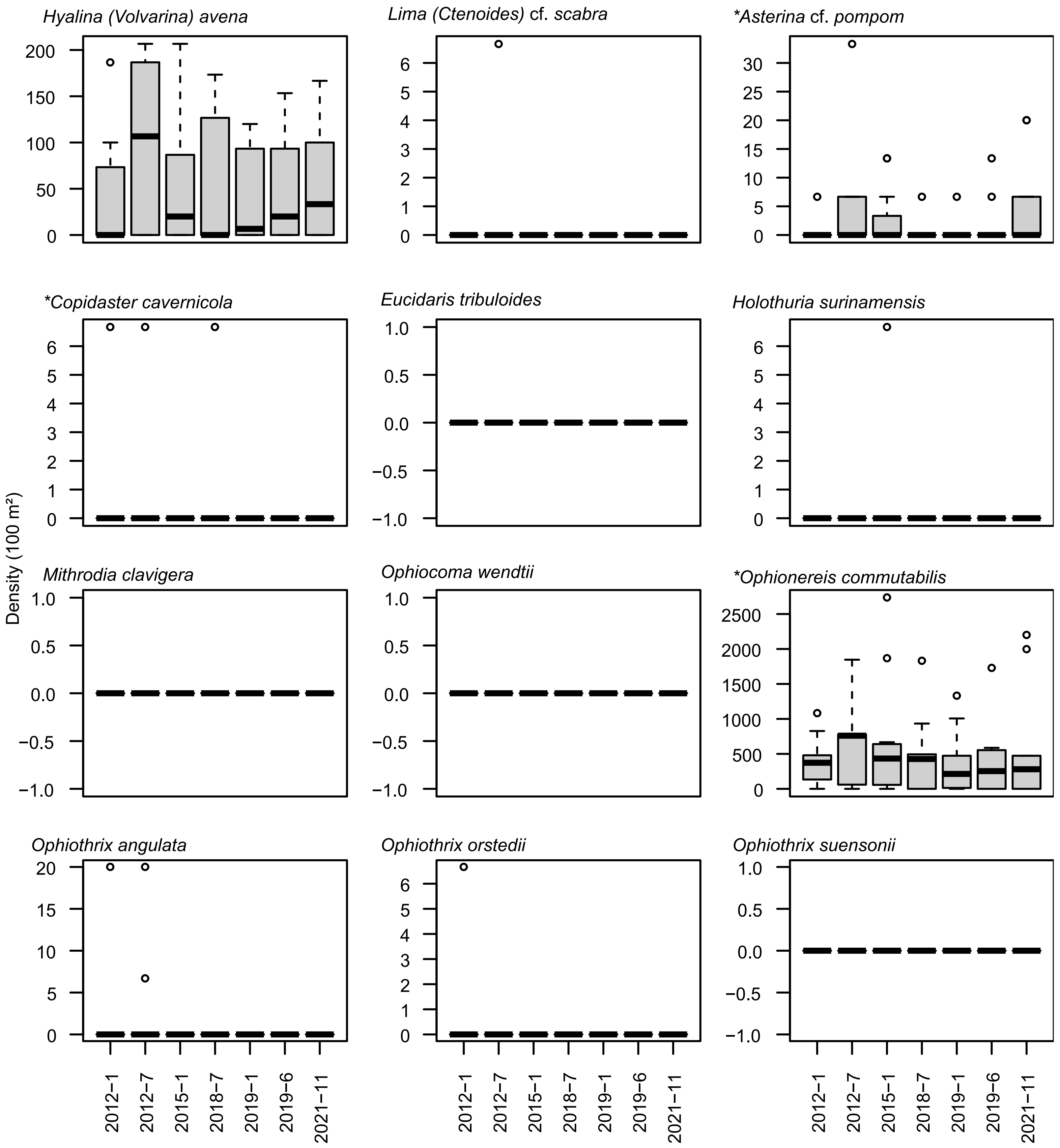

Hyalina (Volvarina) avena
Lima (Ctenoides) cf. scabra
*Asterina cf. pompom
*Copidaster cavernicola
Eucidaris tribuloides
Holothuria surinamensis
Mithrodia clavigera
Ophiocoma wendtii
*Ophionereis commutabilis
Ophiothrix angulata
Ophiothrix orstedii
Ophiothrix suensonii
Density (100 m²)
2012−1
2012−7
2015−1
2018−7
2019−1
2019−6
2021−11

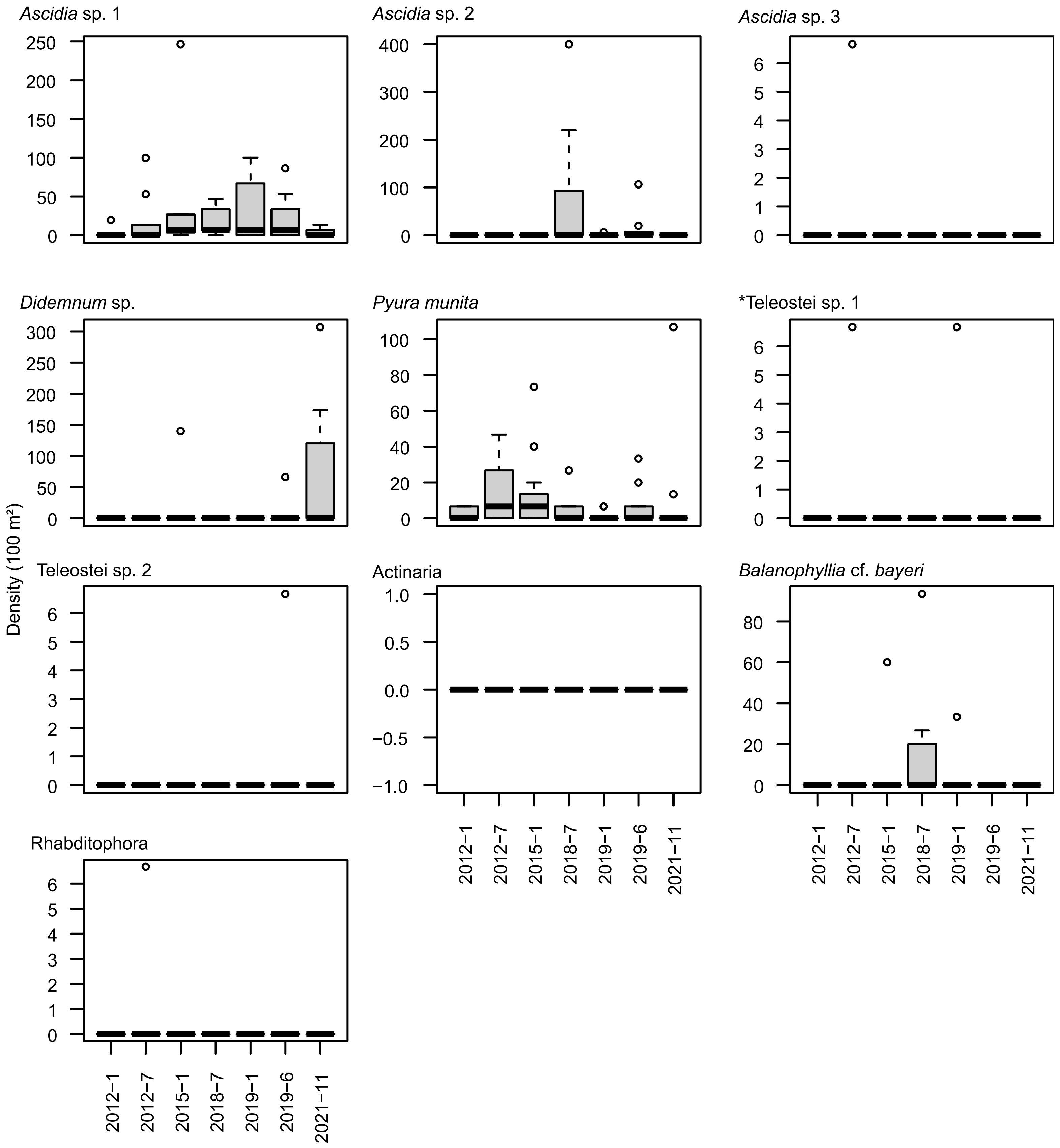
Ascidia sp. 1
Ascidia sp. 2
Ascidia sp. 3
Didemnum sp.
Pyura munita
*Teleostei sp. 1
Teleostei sp. 2
Actinaria
Balanophyllia cf. bayeri
Rhabditophora
Density (100 m²)
2012−1
2012−7
2015−1
2018−7
2019−1
2019−6
2021−11

Densities of the species with at least two individuals recorded in one transect in the site 1 of El Aerolito. (*) Stygobiont.

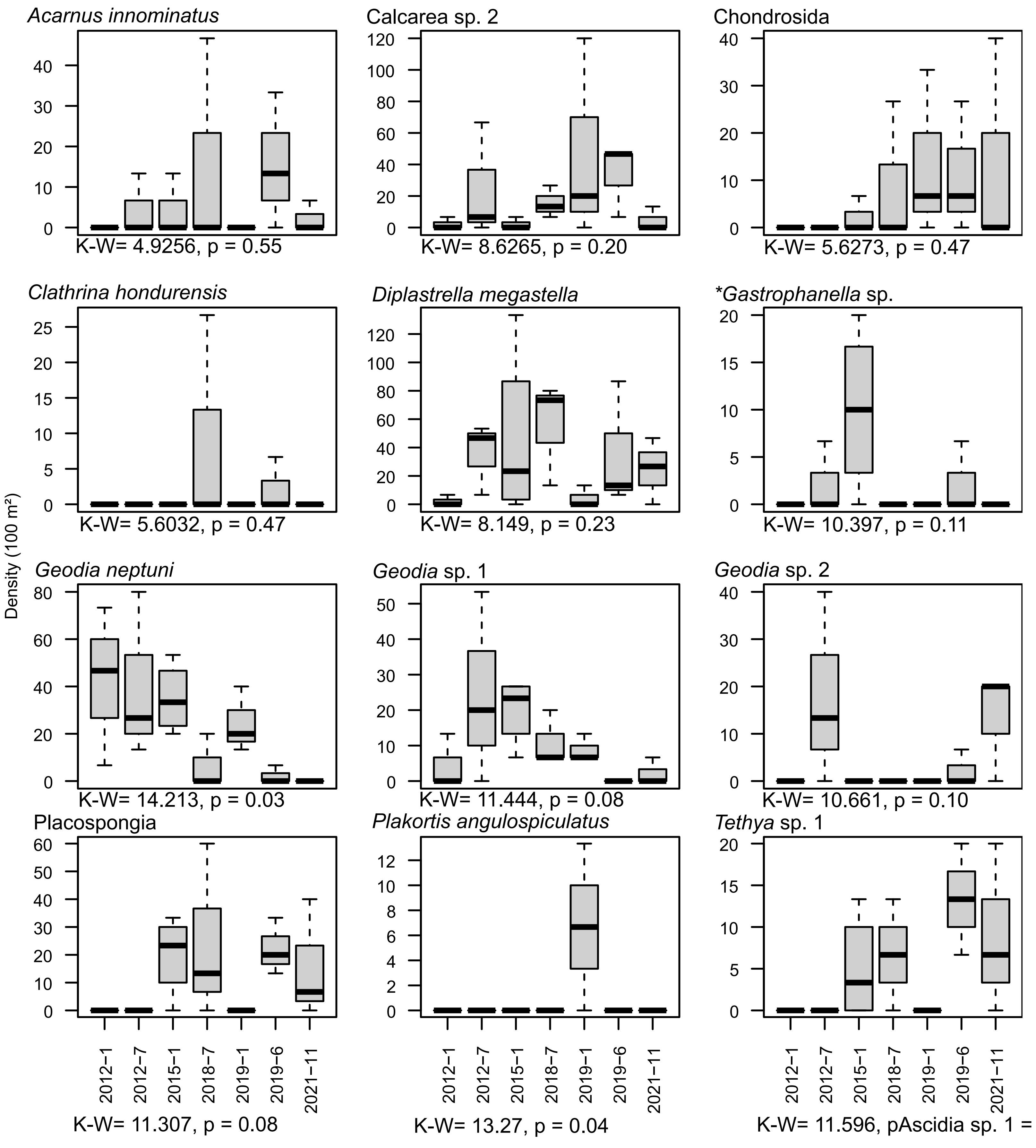

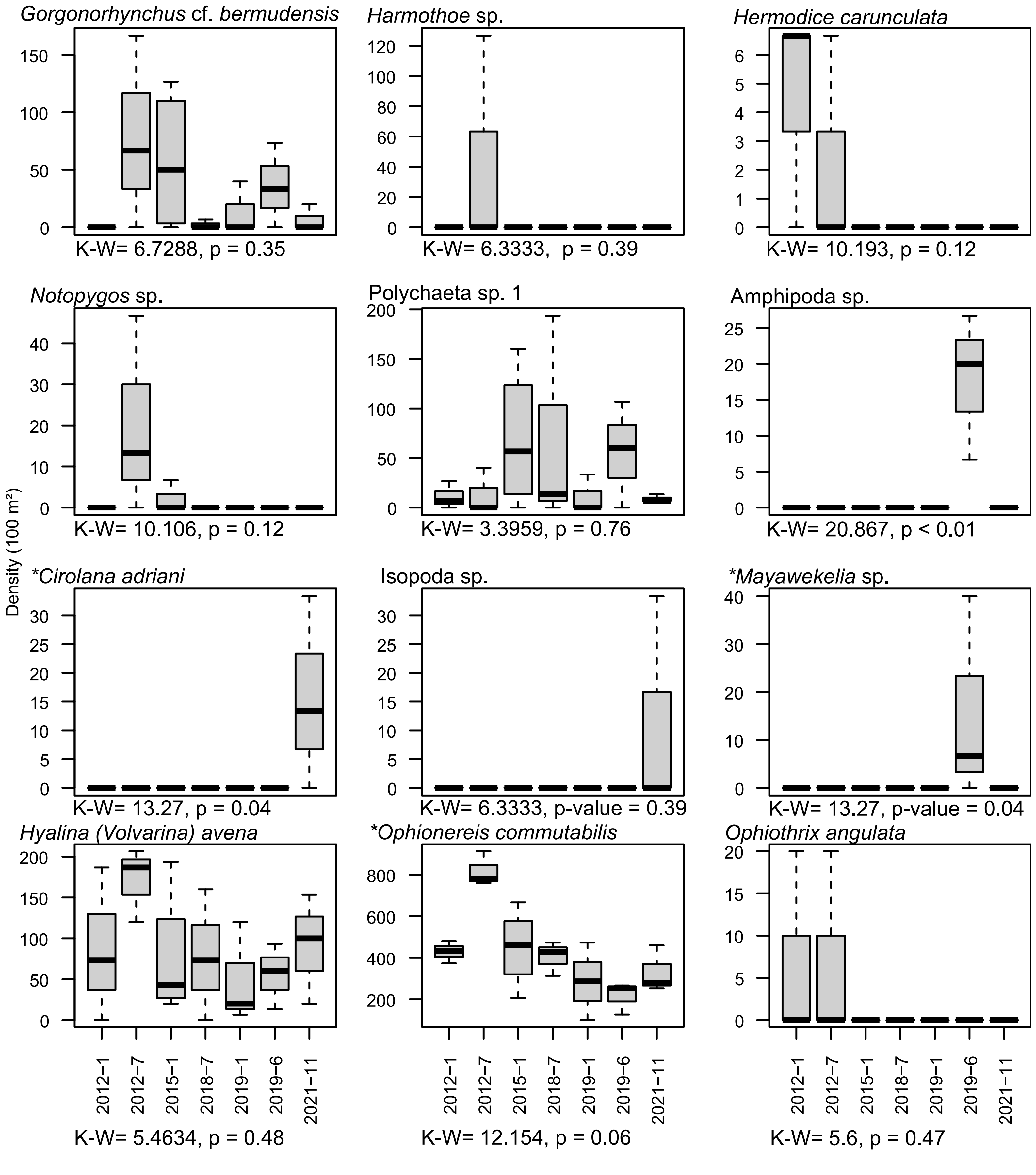
Gorgonorhynchus cf. bermudensis
K-W= 6.7288, p = 0.35
Harmothoe sp.
K-W= 6.3333, p = 0.39
Hermodice carunculata
K-W= 10.193, p = 0.12
Notopygos sp.
K-W= 10.106, p = 0.12
Polychaeta sp. 1
K-W= 3.3959, p = 0.76
Amphipoda sp.
K-W= 20.867, p < 0.01
*Cirolana adriani
K-W= 13.27, p = 0.04
Isopoda sp.
K-W= 6.3333, p-value = 0.39
*Mayawekelia sp.
K-W= 13.27, p-value = 0.04
Hyalina (Volvarina) avena
*Ophionereis commutabilis
Ophiothrix angulata
Density (100 m²)
2012–1
2012–7
2015–1
2018–7
2019–1
2019–6
2021–11
K-W= 5.4634, p = 0.48
K-W= 12.154, p = 0.06
K-W= 5.6, p = 0.47

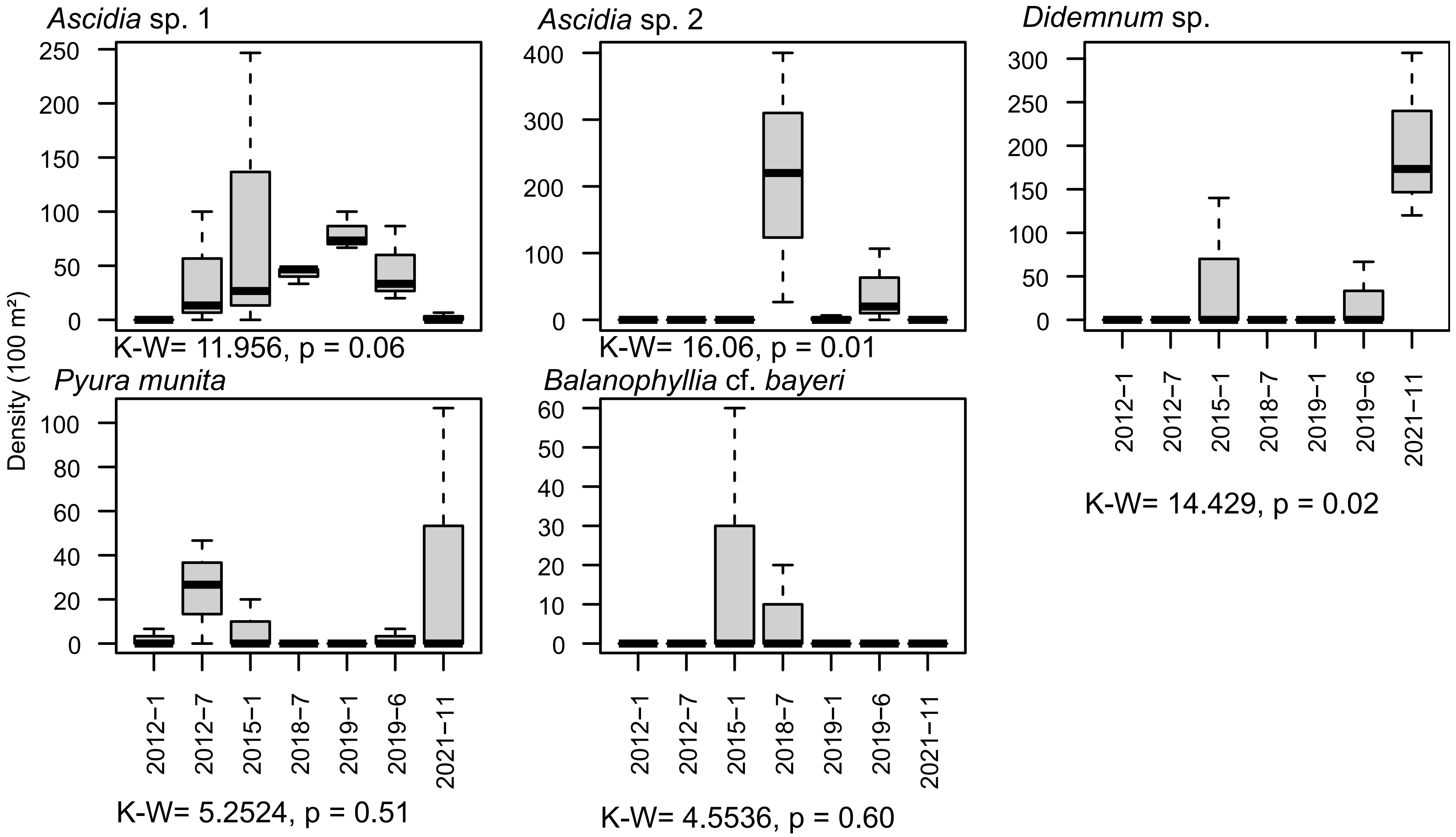
Ascidia sp. 1
K-W= 11.956, p = 0.06
Ascidia sp. 2
K-W= 16.06, p = 0.01
Didemnum sp.
K-W= 14.429, p = 0.02
Pyura munita
K-W= 5.2524, p = 0.51
Balanophyllia cf. bayeri
K-W= 4.5536, p = 0.60
Density (100 m²)
2012–1
2012–7
2015–1
2018–7
2019–1
2019–6
2021–11

Densities of the species with at least two individuals recorded in one transect in the deep section of El Aerolito (site 2).
(*) Stygobiont

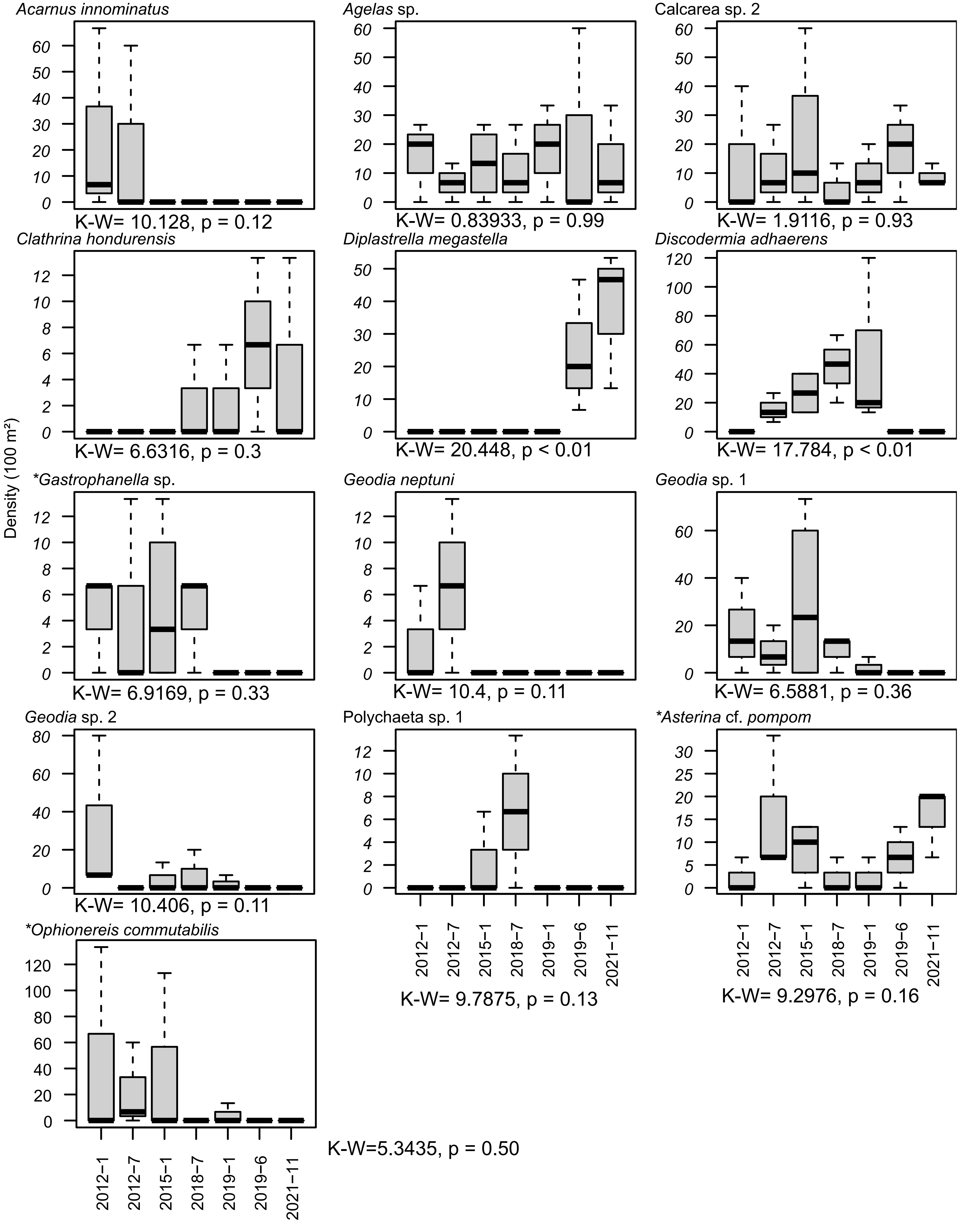

Densities of the species with at least two individuals recorded in one transect in the site 3 of El Aerolito. (*) Stygobiont

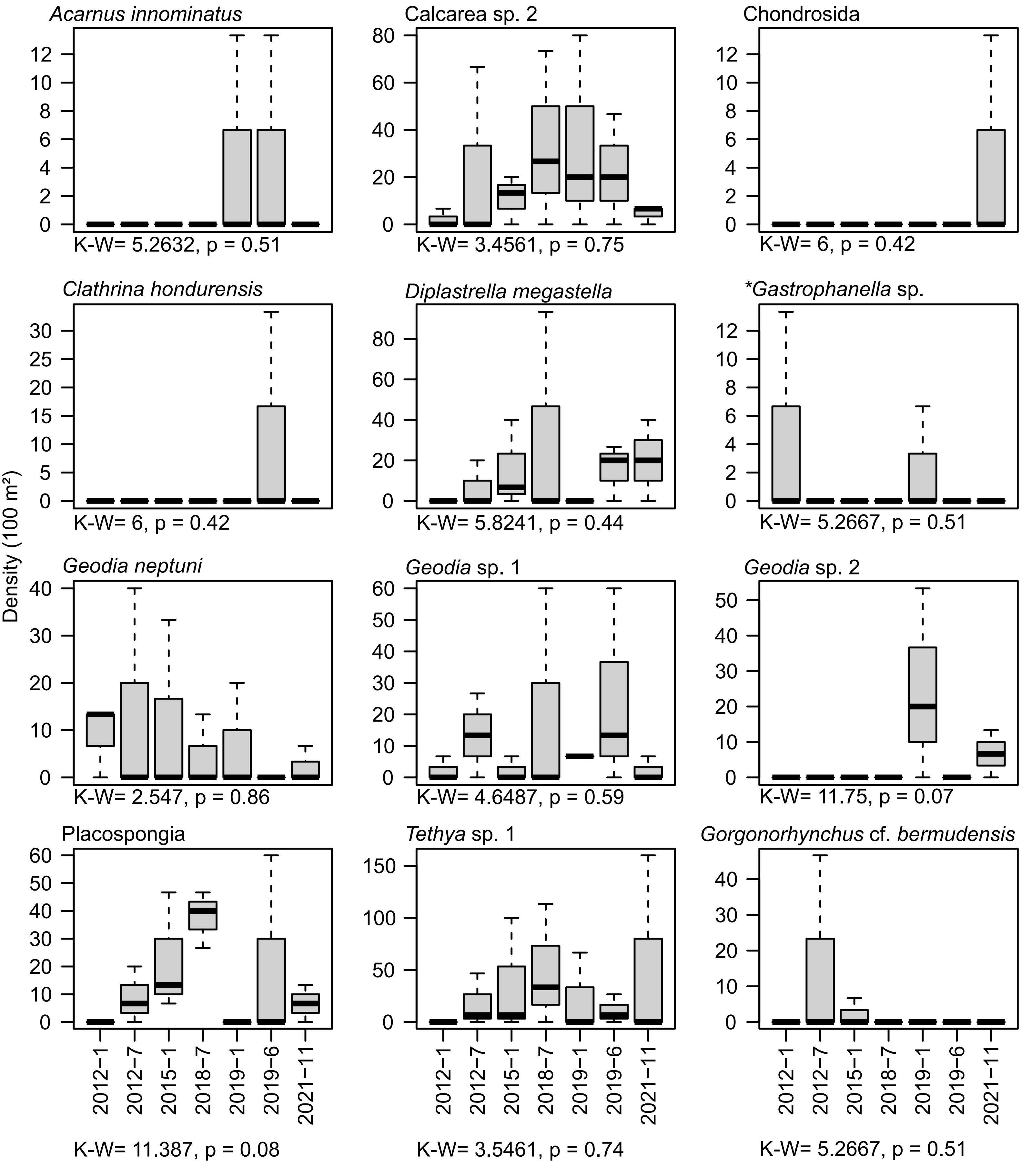

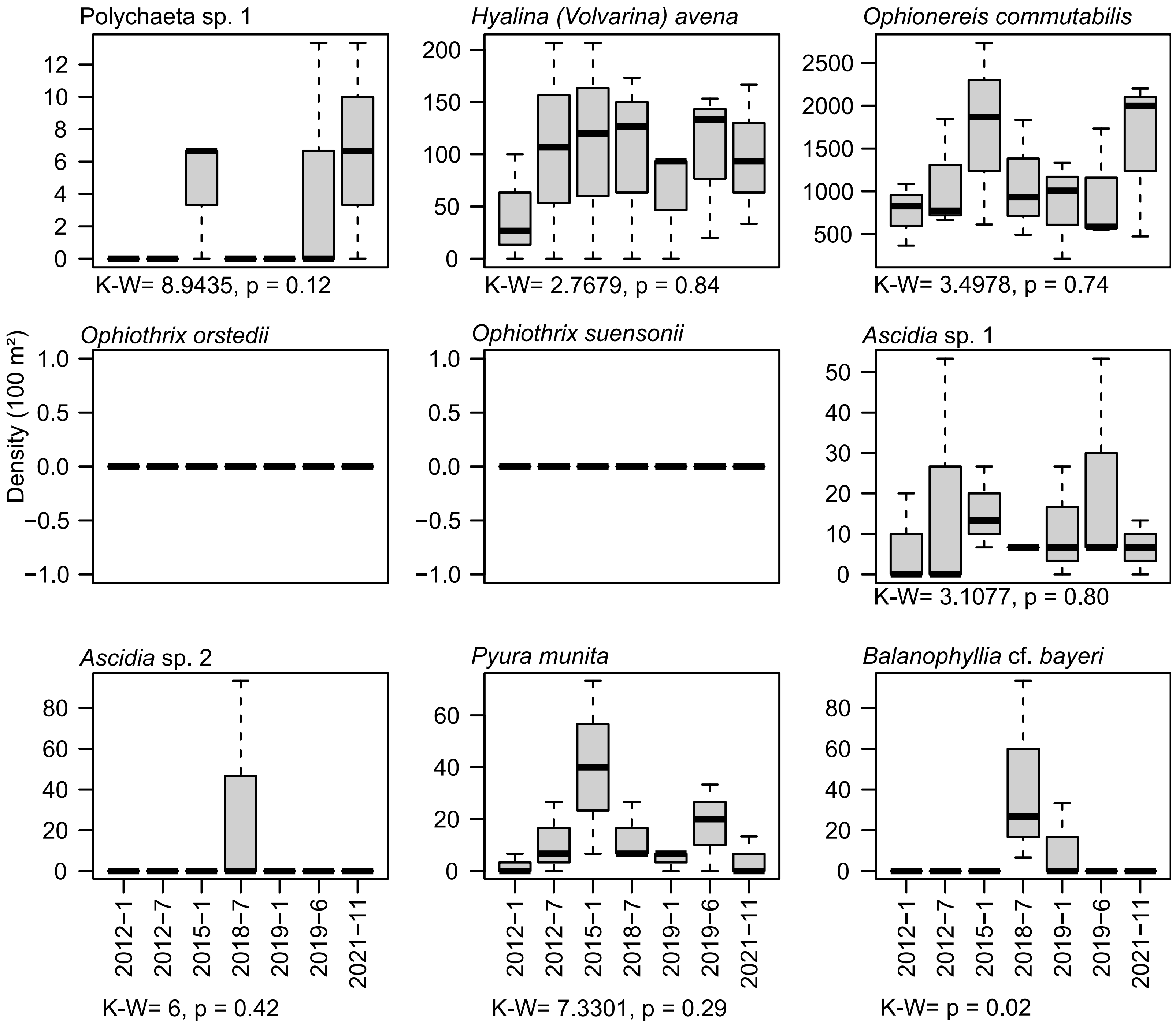
Polychaeta sp. 1
K-W= 8.9435, p = 0.12
Hyalina (Volvarina) avena
K-W= 2.7679, p = 0.84
Ophionereis commutabilis
K-W= 3.4978, p = 0.74
Ophiothrix orstedii
Ophiothrix suensonii
Ascidia sp. 1
K-W= 3.1077, p = 0.80
Density (100 m²)
Ascidia sp. 2
K-W= 6, p = 0.42
Pyura munita
K-W= 7.3301, p = 0.29
Balanophyllia cf. bayeri
K-W= p = 0.02
2012−1
2012−7
2015−1
2018−7
2019−1
2019−6
2021−11
