# Supplementary material for: Response and resilience of karst subterranean estuary communities to precipitation impacts
Source: Ecol Evol. 2023 Aug 14;13(8):e10415. doi: 10.1002/ece3.10415 (PMC10425610; doi:10.1002/ece3.10415)

Supplementary figure 4.- Densities of each species recorded in La Quebrada during the ecological census. (*) Stygobiont

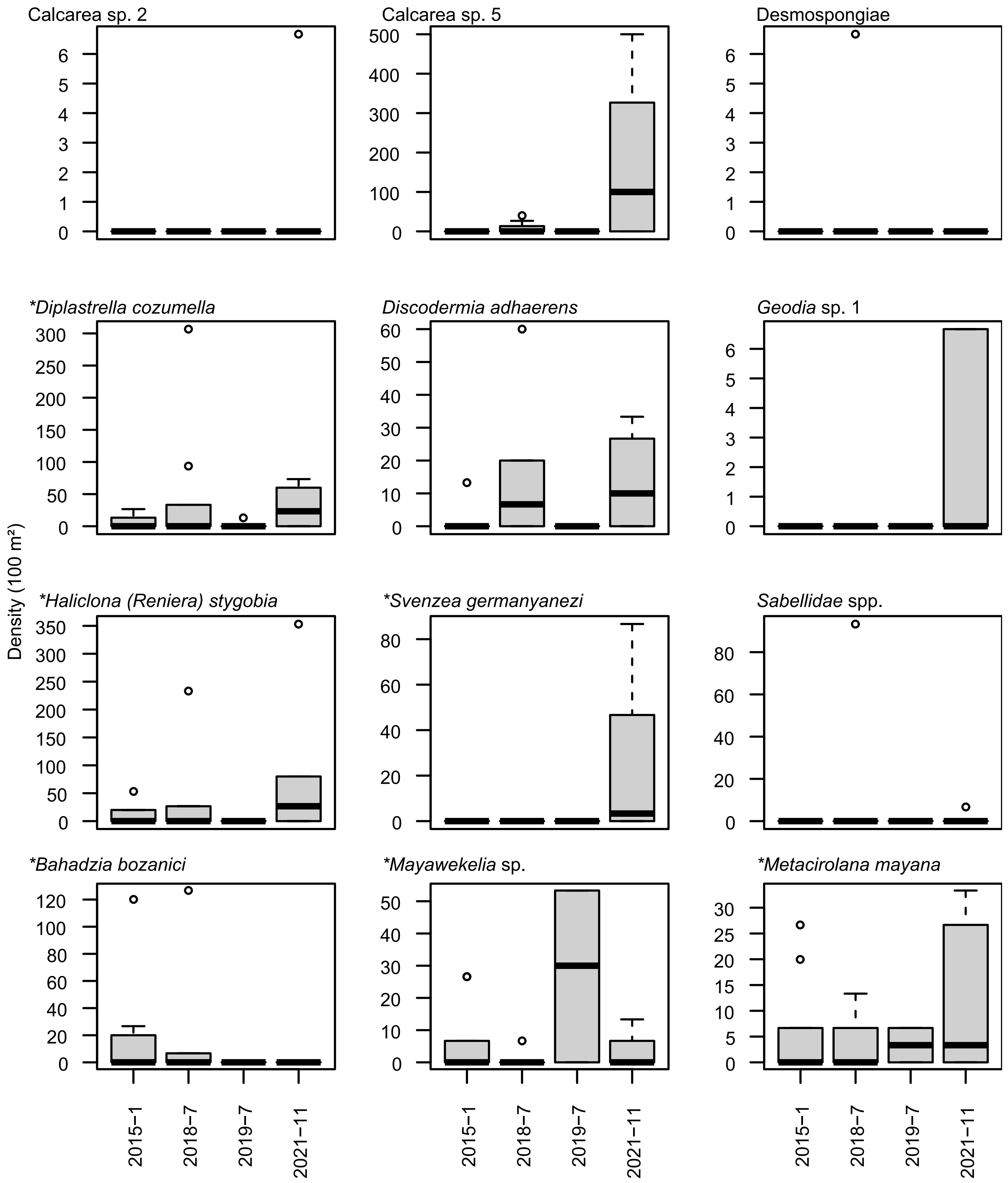

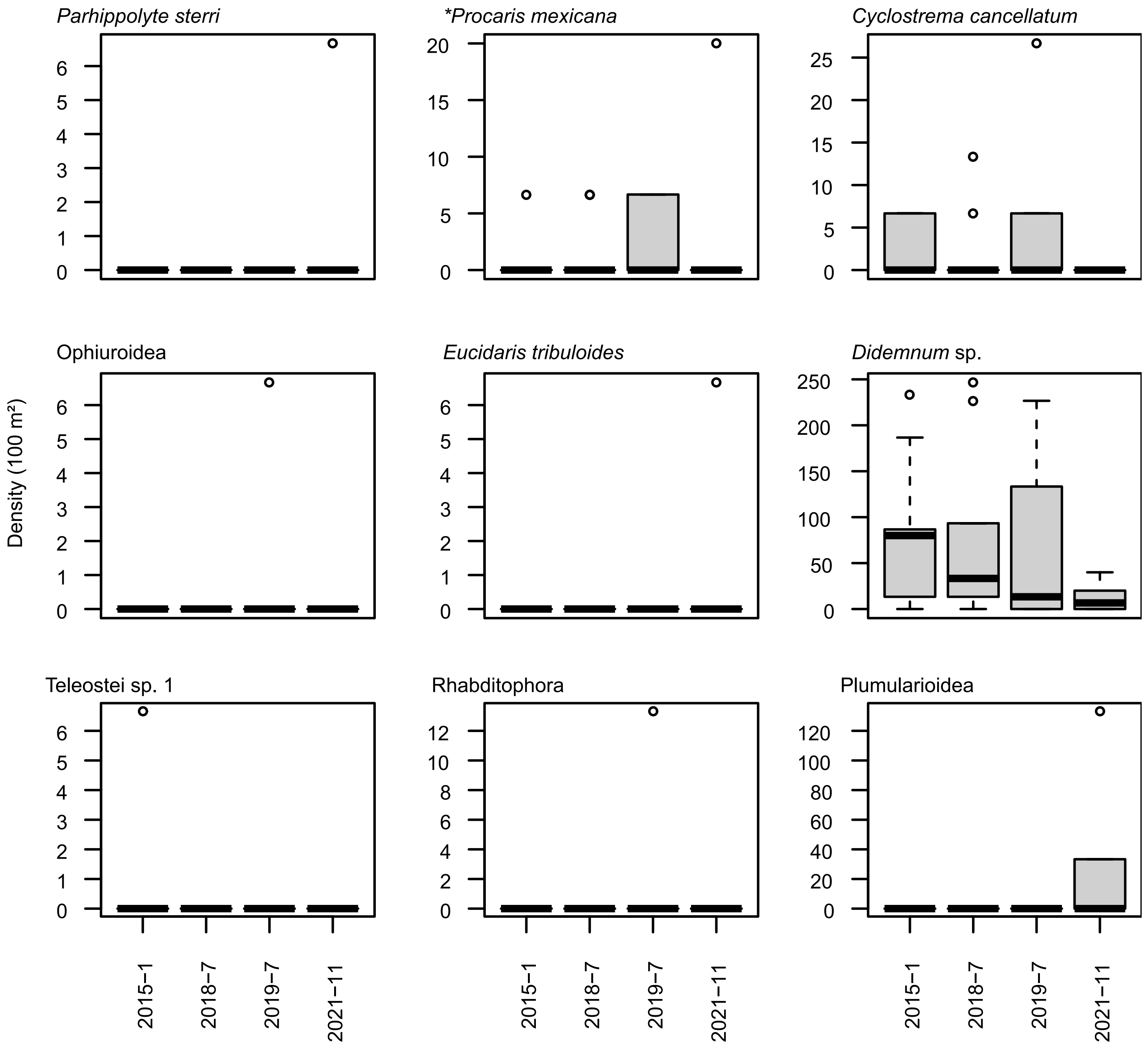

Parhippolyte sterri
*Procaris mexicana
Cyclostrema cancellatum
Ophiuroidea
Eucidaris tribuloides
Didemnum sp.
Teleostei sp. 1
Rhabditophora
Plumularioidea
Density (100 m²)
2015–1
2018–7
2019–7
2021–11

Densities of species with at least two individuals recorded in one transect in the section near the cenote S-1 (site1). (*) Stygobiont

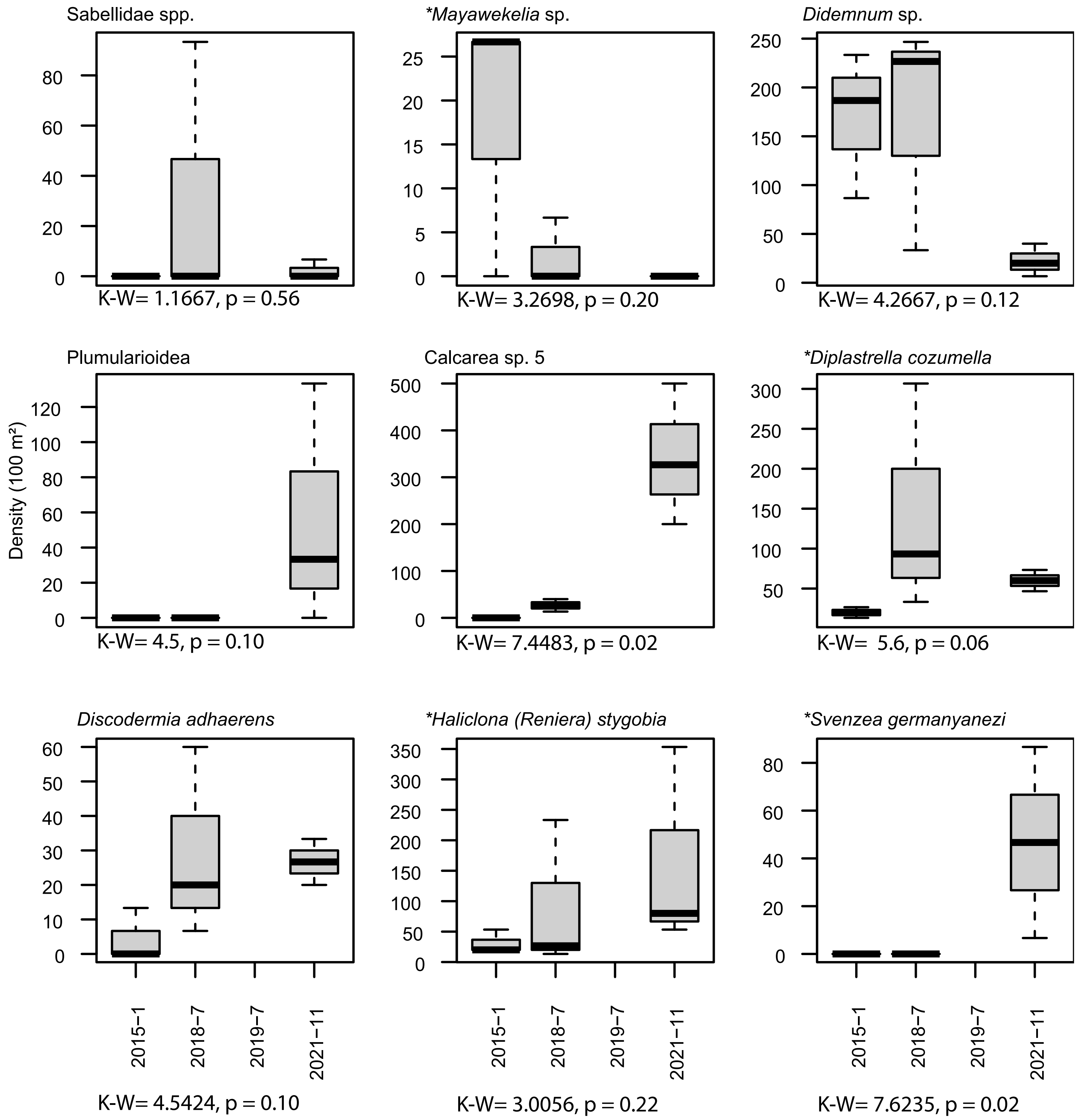

Densities of species with at least two individuals recorded in one transect in the section near the cenote Km1 (site 2).
(*) Sygobiont

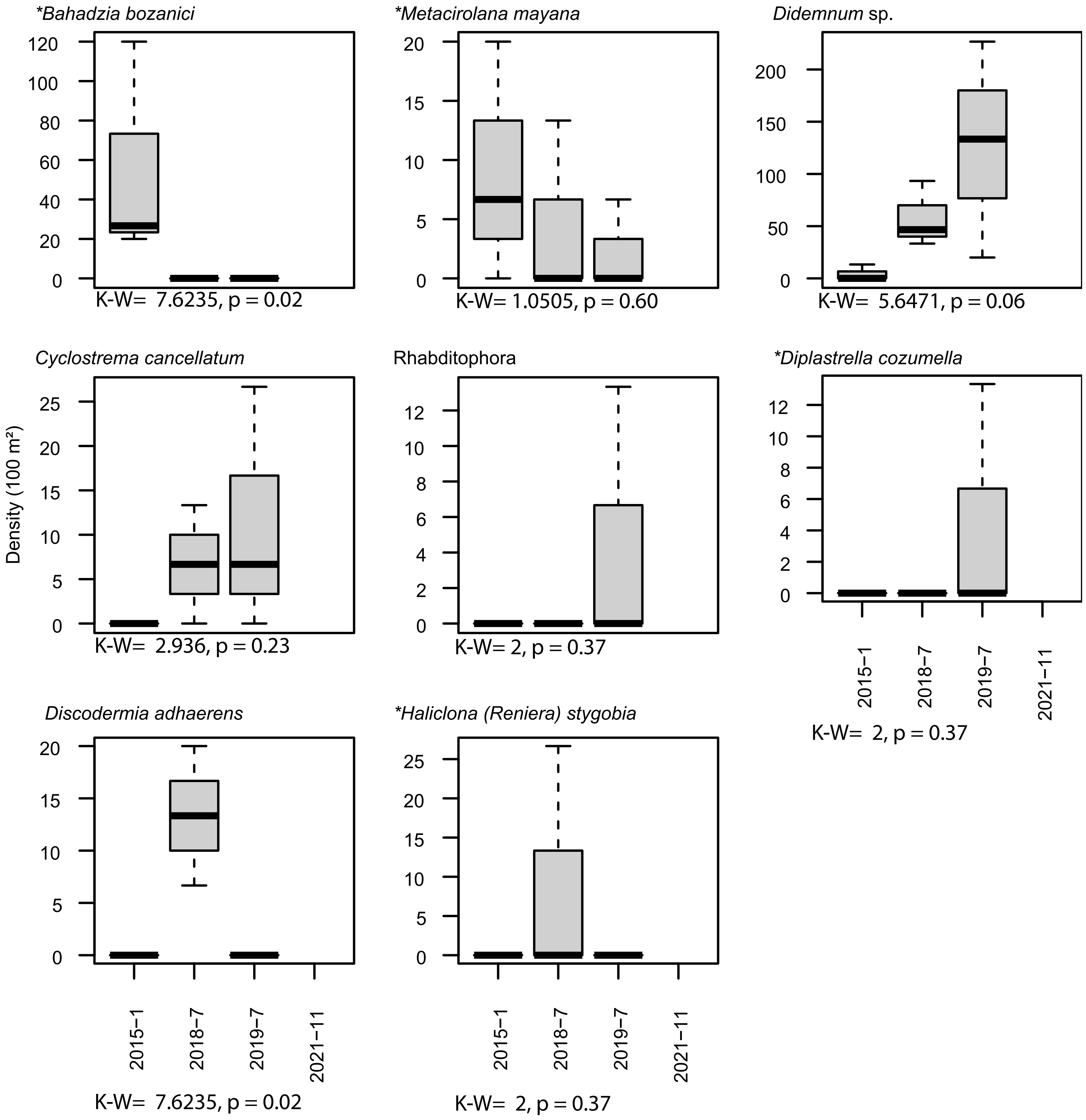

Densities of species with at least two individuals recorded in one transect in the section near the cenote Roca Bomba (site 3).
(*) Stygobiont

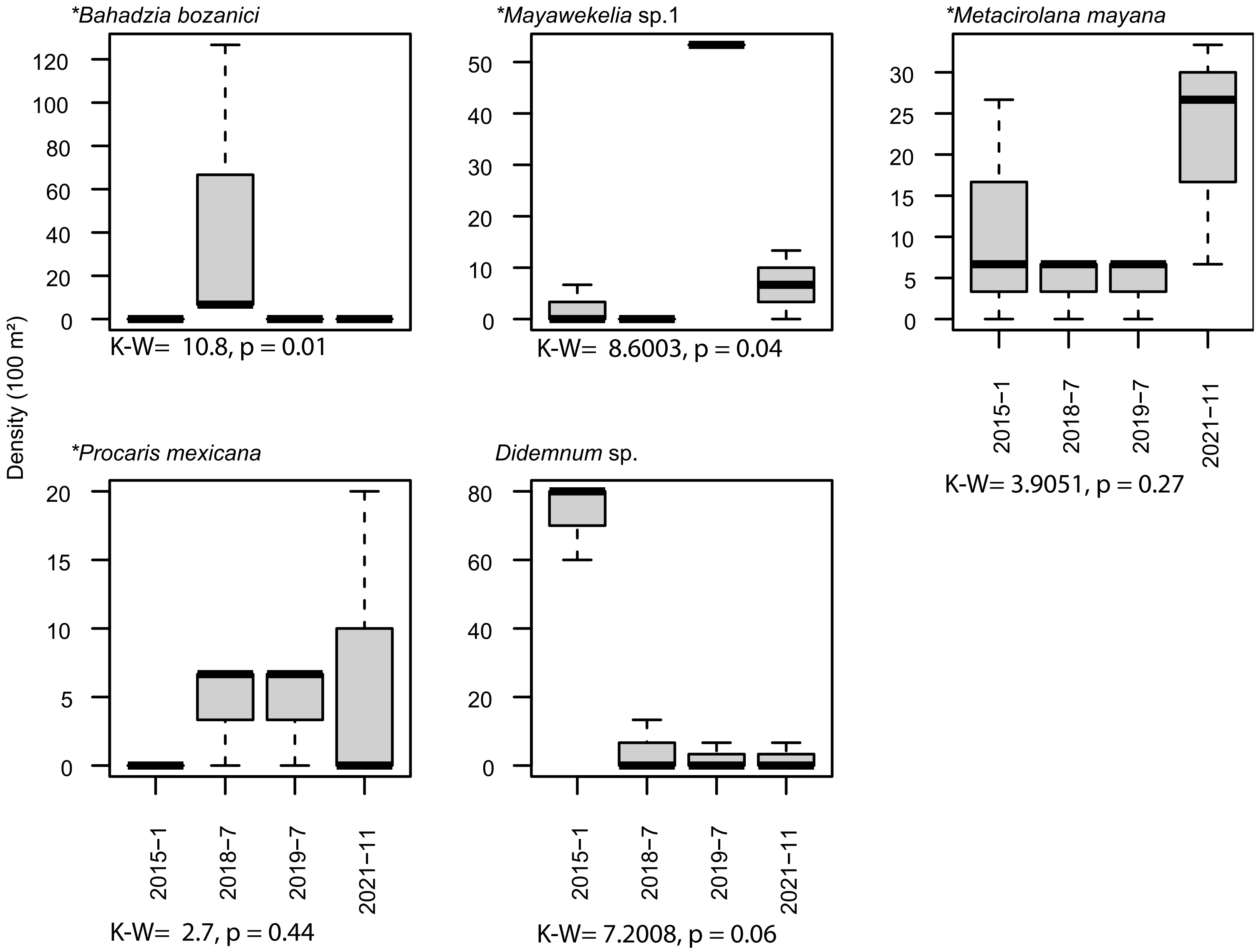

Supplement: Supplementary file 4 — Figure S4 [file ECE3-13-e10415-s002.pdf]
